# Demographic and Socio-Economic Disparities in the Outcomes Among Patients with NVAF Treated with Oral Anticoagulants: A Real-World Evaluation of Medicare Beneficiaries

**DOI:** 10.3390/jcm14093252

**Published:** 2025-05-07

**Authors:** Nipun Atreja, Anandkumar Dubey, Monal Kohli, Jenny Jiang, Melissa Hagan, Gideon Aweh, Shayna Adams, Dong Cheng

**Affiliations:** 1 Bristol Myers Squibb, Lawrenceville, NJ 08648, USA; 2 STATinMED LLC, Dallas, TX 75240, USA

**Keywords:** atrial fibrillation, direct oral anticoagulants, real-world, stroke, switch, systemic embolism, warfarin, socioeconomic

## Abstract

**Objectives**: To assess the association between apixaban use and the risk of stroke/systemic embolism (SE) and major bleeding (MB) compared with other anticoagulants (OACs) across demographic and socio-economic subgroups in the treatment of nonvalvular atrial fibrillation (NVAF). **Methods**: The study included adult NVAF patients initiating OAC treatment between 2013 and 2019 in the Medicare database. Inverse probability treatment weighted Cox proportional hazard models were used to assess stroke/SE and MB outcomes across various subgroups. **Results**: Overall, the adjusted risks of stroke/SE and MB were lower for apixaban compared with warfarin (stroke/SE: HR, 0.69, [95% confidence interval (CI): 0.65–0.74], MB: 0.59 [95% CI: 0.57–0.60]), rivaroxaban (stroke/SE: 0.88 [95% CI: 0.84–0.92], MB: 0.60 [95% CI: 0.58–0.61]) and dabigatran (stroke/SE: 0.88 [95% CI: 0.80–0.95], MB: 0.76 [95% CI: 0.72–0.80]). Among the low socio-economic status (SES) group, apixaban was associated with lower risk vs. warfarin (stroke/SE: 0.73 [95% CI: 0.69–0.77], MB: 0.60 [95% CI: 0.57–0.62]) and rivaroxaban (stroke/SE: 0.88 [95% CI: 0.83–0.94], MB: 0.61 [95% CI: 0.59–0.63]). Among medium SES patients, apixaban was associated with lower risk vs. warfarin (stroke/SE: 0.67 [95% CI: 0.63–0.71] MB: 0.60 [95% CI: 0.58–0.63]), rivaroxaban (stroke/SE: 0.85 [95% CI: 0.79–0.91], MB: 0.59 [95% CI: 0.56–0.61]) and dabigatran (stroke/SE: 0.85 [95% CI: 0.73–0.99], MB: 0.77 [95% CI: 0.70–0.84]). Apixaban was also associated with lower risks of stroke/SE and MB compared with other OACs among most other demographic, socio-economic subgroups. **Conclusions**: Apixaban was associated with lower risk of stroke/SE and MB than warfarin, rivaroxaban, dabigatran across most demographic, socio-economic subgroups.

## 1. Introduction

Atrial fibrillation (AF) is the most common cardiac arrhythmia encountered in clinical practice, with an estimated worldwide prevalence of 59.7 million in 2019 [1]. In the United States (US), both the incidence and prevalence of AF are projected to increase, with the prevalence of AF estimated to be 12 million by 2030 [2]. AF is associated with an increased risk of thromboembolic events such as stroke and systemic embolism (SE) [3,4,5]. AF-related strokes have worse outcomes when compared to strokes due to other causes [6]. Additionally, AF is associated with significantly higher mean total healthcare costs per patient per year than for patients without AF (>$27,000 higher, *p* < 0.001) [7]. Nonvalvular atrial fibrillation (NVAF), defined as AF in the absence of moderate-to-severe mitral stenosis or a mechanical heart valve, is the most prevalent type of AF. In 2018, there were 6.4–7.4 million NVAF diagnoses [8].

Warfarin, a vitamin K antagonist (VKA), has been used for decades for reducing the stroke risk in AF patients [8,9]. It was shown that warfarin users had a 62% reduced risk of stroke than the control arms, including either placebo, aspirin, or other antithrombotic regimens used in clinical practice before 1999 [10]. However, the optimal results of warfarin treatment depend on the maintenance of an international normalized ratio (INR) within a narrow therapeutic range that requires frequent monitoring [11,12]. Failure to maintain the optimal INR may lead to a higher risk of admission to hospital or death due to hemorrhagic or ischemic events [12,13,14,15]. Consequently, patients with poor INR control are also more likely to discontinue warfarin within a year [16,17].

Direct-acting oral anticoagulants (DOACs) are a group of new oral anticoagulants, including direct oral factor Xa inhibitors and direct thrombin inhibitors [8]. Over the past decade, multiple randomized clinical trials have been conducted to compare DOACs with warfarin, demonstrating a favorable efficacy and safety profile for DOACs [18,19,20,21,22]. As a result, DOACs—including apixaban, rivaroxaban, dabigatran, and edoxaban have been approved in the US to reduce the risk of stroke in NVAF patients. Since their approval, several observational studies have also shown that DOAC users were associated with similar or lower risks of stroke/SE and major bleeding compared to warfarin in real-world settings [23,24,25,26,27,28,29]. In addition, DOACs were also associated with lower or comparable healthcare expenditure and/or healthcare utilization than warfarin in other real-world settings [30].

No head-to-head randomized control trials have been published that compare the efficacy and safety profiles among DOACs. However, several retrospective real-world data studies showed that apixaban was associated with a lower risk of stroke/SE and MB compared to rivaroxaban and dabigatran [27,28,31,32].

Patients in the older age group have a higher burden of not only AF but also a higher risk of hemorrhagic complications [33]. Moreover, women with AF have also shown higher rates of stroke than men [34]. These high-risk demographic groups are underrepresented in the medical literature comparing the effectiveness and safety profiles of DOACs with warfarin. Despite the steady increase in DOAC use since their approval and recommendation over warfarin as the first-line therapy for the treatment of NVAF [8,9], there remain disparities in DOAC use across different demographic and socio-economic groups. It was shown that DOAC use was lower among Black, women, and patients with low household incomes [35,36]. Individuals from underrepresented racial and ethnic groups with AF have higher rates of stroke and mortality relative to White patients [37,38]. Furthermore, there is no report comparing the effectiveness and safety outcomes of OACs among various demographic and socio-economic subgroups to date.

The current real-world study was conducted to fill the evidence gap by assessing the associations of the risk of stroke/SE and MB with OAC use across the different demographic and socio-economic subgroups in the Medicare population.

## 2. Methods

### 2.1. Data Source

This retrospective observational study utilized research-identifiable fee-for-service (FFS) Medicare claims data from the US Centers for Medicare and Medicaid Services (CMS). CMS Medicare is the federal health insurance program that comprises ~38 million FFS beneficiaries for people aged ≥65 years, certain younger people with disabilities, and people with end-stage renal disease. The database contains medical and pharmacy claims from 100% national Medicare data, which includes hospital inpatient, outpatient, physician visits, Part D, skilled nursing facility, home health agency, and durable medical equipment claims. All the methods in the current study were carried out per the Declaration of Helsinki. This study used a secondary de-identified database in compliance with the US privacy laws and regulations, i.e., the Health Insurance Portability and Accountability Act (HIPAA) of 1996. This study did not involve interaction with human subjects; the collection, use, or transmittal of individually identifiable data; and does not fall under the regulatory definitions of human subjects research; thus, is exempt from the institutional review board (IRB) review and the requirement to obtain written informed consent from participants, as defined by the US Department of Health and Human Services regulations—45 CFR 46.102(f)(2).

### 2.2. Study Design and Population

The study period was between 1 January 2012, and 31 December 2019, including a 12-month baseline period. Patients were included in this study if they had ≥1 inpatient claim or ≥2 outpatient claims for AF (at least 7 days gap between the 2 outpatient claims), identified using International Classification of Diseases, Ninth/Tenth Revision, Clinical Modification (ICD-9-CM/ICD-10-CM) diagnosis codes (see Table S1, Supplementary Materials). The first AF diagnosis claim date should lie between 1 January 2013, and 31 December 2019. Patients were required to have a prescription for an OAC in this period, on or after the first AF diagnosis, and had 12 months of continuous health plan enrollment with medical and pharmacy benefits before treatment initiation.

**Figure 1:**
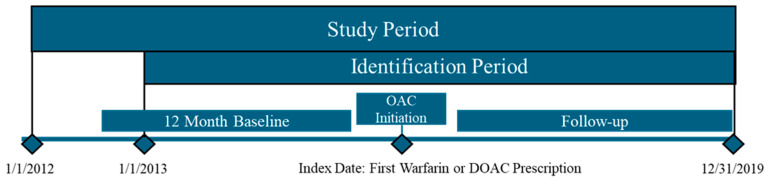
Study design. Abbreviations: DOAC, direct oral anticoagulant; OAC, oral anti-coagulant.

Patients were excluded if they had medical claims indicating a chronic condition, like diagnosis of rheumatic mitral valvular heart disease, venous thromboembolism, transient AF (related to heart valve replacement/transplant, pericarditis, hyperthyroidism, and thyrotoxicity) within the 12 months before or on the index date. Additionally, patients with evidence of pregnancy during the study period were excluded. Other exclusion criteria included recent events such as medical claims indicating hip/knee replacement surgery within six weeks before the index date, pharmacy claims for specific anticoagulants (warfarin, apixaban, rivaroxaban, dabigatran or edoxaban) during the baseline period, and having more than one type of OAC prescription claim on the index date. The index date was defined as the first prescription date of an OAC during the identification period, and the baseline period was defined as 12 months prior to the index date. The follow-up period started from the day after the index date until 30 days after discontinuation of the index OAC, switch to another OAC, death, end of the study period, or health plan disenrollment, whichever occurred earliest. Patients were considered discontinued if there was no extra refill for the index OAC after 30 days of the previous prescription’s run-out date. The discontinuation date was defined as the prescription run-out date. A switch among index OACs was defined as a prescription filled for non-index OAC within 30 days before or after the run-out date. Patients with zero days of follow-up were excluded. Figure 1 denotes a graphical representation of the study design.

### 2.3. Exposure, Outcome, and Covariate Identification

Patients were categorized into four cohorts based on their index prescription of warfarin, apixaban, rivaroxaban, or dabigatran. Due to the low use of edoxaban, it was not further analyzed by this study. The primary outcome events were stroke/SE or MB, identified using ICD-9/10-CM codes in the follow-up period. A stroke/SE event was stratified into three categories: ischemic stroke, hemorrhage stroke, and SE (see Table S2, Supplementary Materials). An MB event was stratified into three categories: gastrointestinal hemorrhage, intracranial hemorrhage, and other bleeding (see Table S3, Supplementary Materials). The time to event was assessed and defined as the number of days from the index date to the first stroke/SE or MB occurrence. The stroke/SE or MB incidence rate was calculated per 100 person-years.

Covariates measured in the baseline period included age, gender, race, geographic region, Medicare/Medicaid dual eligibility, Part D low-income subsidy (LIS), and socio-economic status (SES). Age was classified into 65–74, 75–84, and 85+ years old groups. Race was categorized into White, Black, Asian, and other categories. Geographic regions included Northeast, Midwest, South, West, and Unknown. Medicare/Medicaid dual eligibility was used as a binary variable depending on status in index month and year. Part D low-income subsidy was also a binary variable, and patients were considered for LIS if they received any percentage of Part D subsidy on the index date. SES included low SES if it was within 0–200% of the federal poverty level (FPL), medium SES within 201–400%, and high SES if it was 401% or greater. In addition, baseline clinical variables included the Deyo–Charlson comorbidity index (CCI) score, CHA2DS2-VASc score, HAS-BLED score, history of bleeding, history of stroke/SE, obesity, congestive heart failure, diabetes mellitus, hypertension, chronic obstructive pulmonary disease (COPD), renal disease, myocardial infarction, dyspepsia or stomach discomfort, non-stroke/SE peripheral vascular disease, transient ischemic attack (TIA), coronary artery disease (CAD), baseline medication use such as angiotensin-converting enzyme inhibitors, amiodarone, angiotensin receptor blockers, beta-blockers, H2-receptor antagonists, proton pump inhibitors, anti-platelets, statins, and nonsteroidal anti-inflammatory drugs (NSAIDs). Index DOAC dose which was categorized as standard (apixaban: 5 mg; rivaroxaban: 20 mg; dabigatran: 150 mg), or low dose (apixaban: 2.5 mg; rivaroxaban: 15 mg; dabigatran: 75 mg, 110 mg). All warfarin doses were categorized as standard.

### 2.4. Statistical Analysis

Baseline demographic and clinical characteristics were summarized using means, medians, and standard deviations for continuous variables, and categorical variables were described with frequency counts and percentages. Continuous variables were compared using a *t*-test, whereas categorical variables were compared using a chi-square test between cohorts.

Inverse probability treatment weighting (IPTW) was used to balance patient characteristics and remove confounders when comparing outcomes. IPTW used propensity scores to estimate a treatment’s average effect in the OAC treatment cohorts, conditional on the following observed baseline covariates: age, gender, race, dual eligibility, LIS, SES, index year, baseline comorbidities, and medication use. The propensity score was calculated using a multinomial logistic regression with warfarin users as a reference group. Cox proportional hazard models were used to assess the association of OACs with stroke/SE and MB events. The hazard ratio and 95% confidence intervals (CI) are provided. Statistical analyses were performed using SAS 9.4 (SAS Institute, Cary, NC, USA).

Subgroup analyses were conducted to determine whether the associations of OAC treatment with stroke/SE and MB events differ by demographic or socio-economic groups, with a specific focus on SES.

## 3. Results

**Figure 2:**
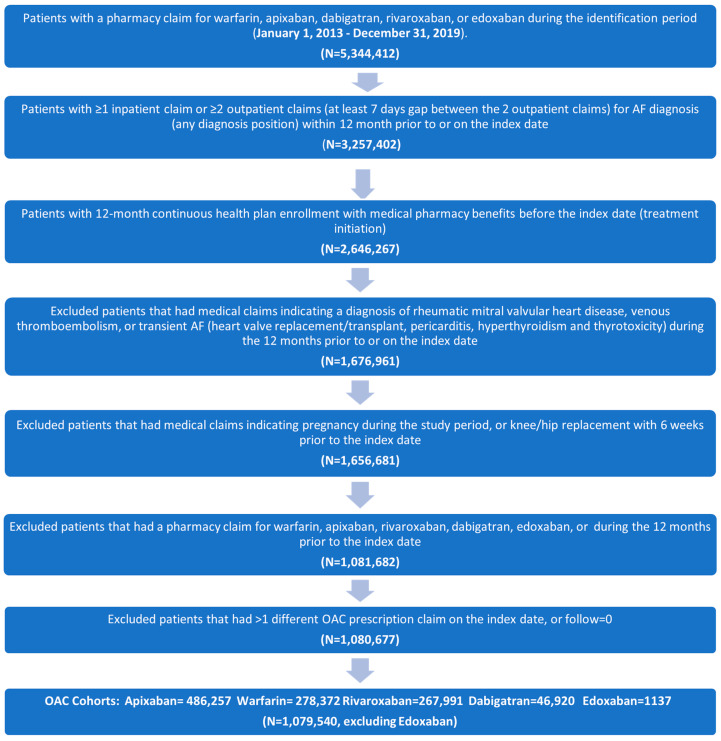
Flow diagram of patients included in the study. Abbreviations: AF, atrial fibrillation; OAC, oral anti-coagulant.

A total of 5,344,412 patients who had claims for any of the OACs of interest during the identification period were identified. After applying the inclusion and exclusion criteria, 1,079,540 eligible patients were included in the final analyses (Figure 2).

### 3.1. Study Cohorts and Baseline Characteristics

There were 278,372 patients in the warfarin cohort, 486,257 in the apixaban cohort, 267,991 in the rivaroxaban cohort, and 46,920 in the dabigatran cohort. The edoxaban cohort was not included in the analysis because of the low sample size (N = 1137). Baseline patient characteristics before and after IPTW are presented in Table 1 and Table 2, respectively.

Homogeneity of patient characteristics across cohorts after IPTW (Table 2) was demonstrated by the standardized difference (STD) values of less than 10% [39]. Most patients in all the weighted cohorts were at least 77 years old, with 78.3–79.2% in the age range 65–84 years, White (88.1–89.5%), and residing in the Southern (37.5–39.3%) or Midwest (23.6–26.3%) region of US at baseline. There was almost an equal proportion of male (47.8–51.4%) to female (48.6–52.2%) patients in all treatment arms. However, patients in the warfarin cohort were more likely to be male (51.4%) and have medium SES (40.9%) compared to all other cohorts. Patients in the rivaroxaban cohort were more likely to receive Part D low-income subsidy (20.6%) and be dual Medicaid eligible (15.4%) than all other cohorts. There were 13.2–15.4% patients with dual Medicaid eligibility and 18.1–20.6% patients with Part D low-income subsidy.

The utilization trend of OACs was similar in all treatment arms between the 2013 and 2019 index years. OAC use increased from 11.7–11.8% in 2013 to 16.0–16.2% in 2018, and then a minor decrease in 2019 (15.5–15.7%). The mean Deyo–Charlson comorbidity index score, CHA_2_DS_2_-VASc score, and HAS-BLED scores were lowest in the apixaban (5.4, 4.5, 2.6) cohort compared to the other cohorts. The mean Deyo–Charlson comorbidity index score was highest in the warfarin (5.5) cohort. The mean CHA_2_DS_2_-VASc and HAS-BLED scores were highest in dabigatran (4.5, 2.6). The most common comorbidities at baseline in all cohorts were hypertension (83.1–85.6%), CAD (41.38–42.7%), and diabetes mellitus (34.5–36.2%). Around 12.0–14.4% had a history of stroke, and 16.7–19.3% had a history of bleeding. The most common medications at baseline in all cohorts were statins (55.2–58.8%) and beta blockers (59.2–63.4%). Most patients in the DOAC cohorts received a standard dose (62.4–73.9%).

The average follow-up time ranged from 335 (dabigatran) to 455 (apixaban) days. Dabigatran patients were least likely to be censored due to death, disenrollment, end of study period, or discontinuation. This was likely because they were the most likely to switch (19.7%) compared to all other cohorts. Switch rates were lower in the apixaban cohort (6.4%) compared to the warfarin (13.8%), dabigatran (19.7%), and rivaroxaban (10.2%) cohorts. Dabigatran patients had the lowest proportion of incident stroke (1.2%), followed by apixaban and rivaroxaban (both approximately 1.3%). Apixaban had the longest time to stroke (376 days) compared to other cohorts. Apixaban had the lowest incidence rate of both stroke/SE (1.1 per 100 person-years) and MB (2.3 per 100 person-years) compared to the other cohorts.

### 3.2. Outcomes

Among the overall cohort, patients on apixaban had a 31% lower risk of stroke (HR: 0.69 [95% confidence interval (CI): 0.65–0.74]; *p* < 0.0001) and 41% lower risk of MB (HR: 0.59 [95% CI: 0.57–0.60]; *p* < 0.0001) than warfarin (Table 3 and Table 4). Patients on dabigatran had a 28% lower risk of stroke (HR: 0.82 [95% CI: 0.69–0.98]; *p* = 0.0254) and 23% lower risk of MB (HR: 0.77 [95% CI: 0.73–0.81]; *p* < 0.0001) than warfarin. Patients on rivaroxaban had a 23% lower risk of stroke (HR: 0.77 [95% CI: 0.71–0.84]; *p* < 0.0001) and a similar risk of MB (HR: 0.99 [95% CI: 0.96–1.01]; *p* = 0.3181) compared to warfarin. Patients on apixaban had a 12% lower risk of stroke (HR: 0.88 [95% CI: 0.84–0.92]; *p* < 0.0001) and 40% lower risk of MB (HR: 0.60 [95% CI: 0.58–0.61]; *p* < 0.0001) than rivaroxaban. Patients on apixaban also had a 12% lower risk of stroke (HR: 0.88 [95% CI: 0.80–0.95]; *p* = 0.0029) and 24% lower risk of MB (HR: 0.76 [95% CI: 0.72–0.80]; *p* < 0.0001) than dabigatran.

When stratified into various demographic subgroups, among females, a 28% lower risk for stroke/SE (HR: 0.73 [95% CI: 0.67–0.78]; *p* < 0.0001) and 40% lower risk for MB (HR: 0.60 [95% CI: 0.58–0.62]; *p* < 0.0001) was observed for apixaban, compared with warfarin. In comparison to rivaroxaban, apixaban was associated with a 12% lower risk of stroke/SE (HR: 0.88 [95% CI: 0.83–0.93]; *p* < 0.0001) and 41% lower risk of MB (HR: 0.59 [95% CI: 0.57–0.61]; *p* < 0.0001). Apixaban was associated with a similar risk of stroke/SE (HR: 0.98 [95% CI: 0.86–1.11]; *p* = 0.7383) and a 28% lower risk of MB (HR: 0.72 [95% CI: 0.66–0.77]; *p* < 0.0001) compared to dabigatran. Among males, the risks of stroke/SE and MB were significantly lower for apixaban compared with warfarin (*p* < 0.0001 for stroke/SE and MB), rivaroxaban (*p* = 0.003 for stroke/SE and *p* < 0.0001 for MB), and dabigatran (*p* = 0.0003 for stroke/SE and *p* < 0.0001 for MB; Table 3 and Table 4).

Among Black patients, the risk was lower for apixaban compared with warfarin (stroke/SE: 0.72 [95% CI: 0.63–0.83] *p* < 0.0001, and MB: 0.59 [95% CI: 0.54–0.65] *p* < 0.0001), also lower risk of MB for apixaban compared with rivaroxaban (MB: 0.56 [95% CI: 0.5–0.62] *p* < 0.0001), but similar risk of stroke/SE compared to rivaroxaban (*p* = 0.2314) and dabigatran (*p* = 0.105), and similar risk of MB compared to dabigatran (*p* = 0.3120). Among White patients, the risk of stroke/SE and MB was significantly lower for apixaban compared with warfarin (both *p* < 0.0001), rivaroxaban (both *p* < 0.0001), and dabigatran (SE *p* = 0.0007, MB *p* < 0.0001; detailed results in Table 3 and Table 4, respectively).

When stratified into various socio-economic subgroups, within the low SES patient subgroup, apixaban was associated with 27% lower risk of stroke (HR: 0.73 [95% CI: 0.69–0.77]; *p* < 0.0001) and 40% lower risk of MB (HR: 0.60 [95% CI: 0.57–0.62]; *p* < 0.0001) than warfarin. Apixaban was associated with a 12% lower risk of stroke (HR: 0.88 [95% CI: 0.83–0.94]; *p* = 0.0001) and 39% lower risk of MB (HR: 0.61 [95% CI: 0.59–0.63]; *p* < 0.0001) than rivaroxaban and a 24% lower risk of MB (HR: 0.76 [95% CI: 0.70–0.83]; *p* < 0.0001) than dabigatran.

Within medium SES patients, apixaban was associated with 33% lower risk of stroke (HR: 0.67 [95% CI: 0.63–0.71]; *p* < 0.0001) and 40% lower risk of MB (HR: 0.60 [95% CI: 0.58–0.63]; *p* < 0.0001) than warfarin. Apixaban was associated with 15% lower risk of stroke (HR: 0.85 [95% CI: 0.79–0.91]; *p* < 0.0001) and a 41% lower risk of MB (HR: 0.59 [95% CI: 0.56–0.61]; *p* < 0.0001) compared to rivaroxaban; and a 15% lower risk of stroke (HR: 0.85 [95% CI: 0.73–0.98]; *p* = 0.0231) and 23% lower risk of MB (HR: 0.77 [95% CI: 0.70–0.84]; *p* < 0.0001) than dabigatran.

Within high SES patients, apixaban was associated with 32% lower risk of stroke (HR: 0.68 [95% CI: 0.62–0.74]; *p* < 0.0001) and 44% lower risk of MB (HR: 0.56 [95% CI: 0.52–0.59]; *p* < 0.0001) than warfarin. Apixaban was associated with a 27% lower risk of stroke (HR: 0.73 [95% CI: 0.61–0.87]; *p* = 0.0006) and a 25% lower risk of MB (HR: 0.75 [95% CI: 0.66–0.85]; *p* < 0.0001) compared to dabigatran. Apixaban was associated with a 41% lower risk of MB (HR: 0.59 [95% CI: 0.55–0.62]; *p* < 0.0001) than rivaroxaban.

## 4. Discussion

The current study used real-world data to assess the risk of stroke/SE and MB associated with OACs within various demographic and socio-economic subgroups in the Medicare population. In particular, this study included previously underexplored patient populations, such as female, Black populations and patients of lower incomes defined by SES status and Medicare/Medicaid dual eligibility. Apixaban was associated with lower rates of stroke/SE and MB compared to other OACs across most strata and vulnerable patient populations. The final cohort included over one million patients. Most were White, residing in the Southern or Midwest region of the US, and about 40% belonged to underserved populations, denoted by the low SES group.

During the study period, utilization of apixaban steadily increased, rivaroxaban usage was relatively stable, and an overall decreasing trend was observed for warfarin and dabigatran. Less frequent laboratory monitoring, a more predictable pharmacokinetic (dosing) profile, fewer interactions with other drugs, and the non-inferior effectiveness and safety profile of DOACs may explain the increasing DOAC adoption trend in recent years [40,41].

All DOACs were associated with a lower risk of stroke/SE and MB compared to warfarin, except for rivaroxaban, which had a similar risk of MB compared to warfarin. This finding is consistent with a prior Bayesian network meta-analysis that summarized results from 53 real-world studies. Based on that meta-analysis, the use of apixaban, dabigatran, and edoxaban, but not rivaroxaban, was associated with a reduced risk of MB [42].

In the current study, apixaban use was associated with a lower risk of stroke/SE and MB compared to rivaroxaban, dabigatran, and warfarin in the overall study cohort. This result further confirmed a pivotal randomized clinical trial on apixaban [18], similar to the ARISTOPHANES study, wherein apixaban use was associated with about 40% lower risk of stroke/SE and MB than warfarin [27]. Ray et al. also noted that apixaban users had a lower risk of the composite of stroke/SE and MB compared to rivaroxaban [31], and the results from the current study echoed those earlier findings.

While DOACs are effective in reducing the risk of both stroke/SE and MB, there were differences in DOAC use between the different sociodemographic and socio-economic groups. Prior studies found that patients of Black race, women, the elderly, and with low household income were associated with low use of DOACs [35,36,43,44]. Furthermore, patients from underrepresented racial and ethnic groups with AF had higher rates of stroke and mortality [38,40]. These disparities may have profound implications for patients, particularly those from racial and ethnic minorities, women, and the underserved patient population [35,36,37,38,45]. Our study observed that apixaban was associated with a 20–40% lower risk of stroke/SE compared to warfarin across all subgroups. When compared with dabigatran, apixaban was also associated with a lower risk of stroke/SE among males, White patients, patients with medium and high SES, and those who were not Medicare/Medicaid dual eligible. In the remaining subgroups, apixaban use was as effective as dabigatran against stroke/SE. In comparison to rivaroxaban, apixaban also had a lower risk for stroke/SE among the majority subgroups and similar effectiveness among Black and Other race patients, those with high SES levels, and patients with Medicare/Medicaid dual eligibility.

For the MB safety outcome, apixaban was associated with a 40–50% lower risk compared to warfarin across all subgroups. Compared to dabigatran and rivaroxaban, apixaban was also associated with a lower risk of MB across most of the subgroups except in the 65–74 age group, racial subgroups other than White patients, and among the high SES group, where apixaban had a similar risk of MB as with dabigatran and rivaroxaban. The results for apixaban vs. warfarin among the overall study sample, age and gender subgroups were similar to the ARISTOPHANES study [27]. However, the remaining socio-economic subgroups in our study were not assessed in the ARISTOPHANES study.

Taken together, our study found differences in stroke/SE and MB risks depending on the OAC used, and those differences persisted across different age groups, genders, races, SES categories, and Medicare/Medicaid dual eligibility categories. Specifically, in the underserved patient population, denoted by the low SES group, apixaban showed a significantly lower risk of MB as compared to warfarin, rivaroxaban, and dabigatran and a significantly lower risk of stroke/SE compared to warfarin and rivaroxaban. These consistent results obtained from the large healthcare administrative databases help to strengthen the value of apixaban and its benefits across the demographic and socio-economic subgroups.

The current study has several strengths. First, the US CMS Medicare is a federal health insurance program for people aged ≥65 years. Therefore, the results may have greater generalizability among the US population aged 65 years or above. Second, the sample size is over one million, allowing us to conduct multiple subgroup analyses with a relatively large sample size within each subgroup. Third, a robust statistical methodology was used to account for confounding and included a large number of potential covariates in the model to reduce the possibility of residual confounding. However, the results should be interpreted in the context of the following potential limitations. First, due to the observational nature of this study, residual confounding is possible. However, IPTW was used to minimize the effect of residual confounding—by balancing patient characteristics and removing confounders when comparing outcomes. Second, exposure was defined based on pharmacy fills, and filling a prescription does not necessarily mean the medication was consumed, and this could have resulted in exposure misclassification. However, this misclassification is possible across all cohorts and is likely non-differential. Therefore, the results were likely conservative. Third, study outcomes were identified using ICD, CPT, and HCPCS codes and therefore, it is possible to have outcome misclassification due to coding error or data entry errors. Again, this misclassification is also likely a non-differential misclassification. Fourth, SES status was based upon the FPL cut-off on median household income and may have misclassified some patients as this was a proxy variable. Fifth, it should also be noted that unobserved heterogeneity may exist across the datasets used in this analysis. However, the likelihood of duplicate observations is low, researched to be 0.5%, and not likely to significantly impact study results. Sixth, while competing risks, such as treatment switching, were present, we did not employ competing risk analyses, which may overestimate event rates in groups with higher switching. However, given the low overall switching rates (pre-IPTW 9.3%; post-IPTW 9.8%), we anticipate minimal impact on between-group risk comparisons. Finally, edoxaban was excluded due to low sample size during the study period, though its increasing use in recent years warrants future investigation. Additionally, dose-specific effects could not be assessed due to sample size constraints; future studies should explore this.

## 5. Conclusions

The current study found that in the Medicare population, apixaban was associated with lower risks of stroke/SE and MB when compared to warfarin, rivaroxaban, and dabigatran. Importantly, apixaban use was associated with a 20–40% lower risk of stroke/SE and a 40–50% lower MB risk compared to warfarin across most demographic and socio-economic subgroups. These results highlight the effectiveness of apixaban in reducing the risk of stroke/SE with reduced major bleeding risk across diverse demographic and socio-economic groups in real-world settings, especially among female and Black populations and those in low SES groups.

## Figures and Tables

**Table 1:** Baseline demographic, socio-economic and clinical characteristics before IPTW weighting.

	Warfarin Cohort (Reference)		Apixaban Cohort			Dabigatran Cohort			Rivaroxaban Cohort		
	N/Mean	%/SD	N/Mean	%/SD	STD	N/Mean	%/SD	STD	N/Mean	%/SD	STD
**Sample Size**	278,372		486,257			46,920			267,991		
**Age**	78.15	7.59	78.24	7.67	1.19	76.67	7.24	19.97	76.99	7.34	15.50
**65–74**	100,171	35.98%	175,275	36.05%	0.13	20,410	43.50%	15.40	112,551	42.00%	12.35
**75–84**	115,344	41.44%	199,405	41.01%	0.87	18,772	40.01%	2.90	108,354	40.43%	2.04
**≥85**	62,857	22.58%	111,577	22.95%	0.87	7738	16.49%	15.40	47,086	17.57%	12.53
**Gender**											
**Male**	137,785	49.50%	229,549	47.21%	4.58	24,206	51.59%	4.19	135,442	50.54%	2.09
**Female**	140,587	50.50%	256,708	52.79%	4.58	22,714	48.41%	4.19	132,549	49.46%	2.09
**Race**											
**White**	247,642	88.96%	433,127	89.07%	0.36	41,750	88.98%	0.07	238,861	89.13%	0.54
**Black**	16,288	5.85%	23,905	4.92%	4.14	2049	4.37%	6.74	11,971	4.47%	6.26
**Asian**	4094	1.47%	8541	1.76%	2.27	1020	2.17%	5.26	5206	1.94%	3.64
**Other**	10,348	3.72%	20,684	4.25%	2.74	2101	4.48%	3.84	11,953	4.46%	3.75
**U.S. Geographic Region**											
**Northeast**	52,501	18.86%	90,839	18.68%	0.46	9729	20.74%	4.707422	50,053	18.68%	0.47
**Midwest**	90,695	32.58%	111,181	22.86%	21.83	10,597	22.59%	22.50522	63,039	23.52%	20.26
**South**	84,983	30.53%	199,408	41.01%	22.00	17,913	38.18%	16.15975	101,971	38.05%	15.90
**West**	49,498	17.78%	83,439	17.16%	1.64	8551	18.22%	1.15405	52,191	19.47%	4.35
**Other**	695	0.25%	1390	0.29%	0.70	130	0.28%	0.534644	737	0.28%	0.50
**Index Year**											
**2013**	70,654	25.38%	7975	1.64%	74.06	13,330	28.41%	6.83	34,205	12.76%	32.54
**2014**	57,600	20.69%	32,375	6.66%	41.72	8778	18.71%	4.99	44,960	16.78%	10.05
**2015**	46,697	16.78%	56,938	11.71%	14.53	5715	12.18%	13.09	37,579	14.02%	7.63
**2016**	38,021	13.66%	78,297	16.10%	6.87	8109	17.28%	10.03	36,575	13.65%	0.03
**2017**	29,502	10.60%	79,514	16.35%	16.91	6269	13.36%	8.52	44,270	16.52%	17.36
**2018**	20,903	7.51%	111,526	22.94%	43.97	3223	6.87%	2.48	38,304	14.29%	21.90
**2019**	14,995	5.39%	119,632	24.60%	55.88	1496	3.19%	10.87	32,098	11.98%	23.57
**Medicaid Dual Eligibility**	43,345	15.57%	67,727	13.93%	4.63	6501	13.86%	4.84	38,733	14.45%	3.13
**Part-D LIS**	58,494	21.01%	93,537	19.24%	4.43	9074	19.34%	4.17	52,340	19.53%	3.69
**SES Variable**											
**Low**	119,236	42.83%	192,289	39.54%	6.69	18,726	39.91%	5.94	105,076	39.21%	7.37
**Medium**	115,662	41.55%	194,913	40.08%	2.98	18,746	39.95%	3.25	106,738	39.83%	3.50
**High**	43,474	15.62%	99,055	20.37%	12.40	9448	20.14%	11.82	56,177	20.96%	13.86
**Baseline Comorbidity**											
**CCI**	5.65	2.36	5.58	2.39	3.02	5.09	2.21	24.55	5.18	2.26	20.27
**CHA_2_DS_2_-VASc Score**	4.67	1.73	4.51	1.69	9.57	4.27	1.69	23.79	4.25	1.68	24.58
**1**	5077	1.82%	9020	1.85%	0.23	1321	2.82%	6.59	7219	2.69%	5.86
**2**	22,232	7.99%	45,988	9.46%	5.22	5525	11.78%	12.72	32,266	12.04%	13.53
**3**	47,152	16.94%	89,607	18.43%	3.90	9924	21.15%	10.74	56,244	20.99%	10.34
**4+**	203,911	73.25%	341,642	70.26%	6.65	30,150	64.26%	19.49	172,262	64.28%	19.45
**HAS-BLED Score**	2.68	1.22	2.67	1.18	0.76	2.46	1.09	18.66	2.49	1.11	15.81
**0**	8252	2.96%	13,404	2.76%	1.25	1327	2.83%	0.81	8136	3.04%	0.42
**1**	33,793	12.14%	52,770	10.85%	4.04	5935	12.65%	1.55	33,192	12.39%	0.75
**2**	90,995	32.69%	167,862	34.52%	3.88	19,362	41.27%	17.84	105,860	39.50%	14.22
**3+**	145,332	52.21%	252,221	51.87%	0.68	20,296	43.26%	17.99	120,803	45.08%	14.30
**Bleeding history**	53,070	19.06%	83,550	17.18%	4.89	7348	15.66%	8.99	42,634	15.91%	8.31
**Obesity**	56,148	20.17%	124,138	25.53%	12.79	9508	20.26%	0.23	61,321	22.88%	6.60
**CHF**	94,706	34.02%	133,603	27.48%	14.22	11,750	25.04%	19.78	64,648	24.12%	21.93
**DM**	108,007	38.80%	168,146	34.58%	8.76	16,366	34.88%	8.13	90,102	33.62%	10.79
**Hypertension**	234,996	84.42%	413,638	85.07%	1.80	39,553	84.30%	0.33	224,978	83.95%	1.28
**COPD**	84,227	30.26%	141,165	29.03%	2.68	12,764	27.20%	6.75	74,065	27.64%	5.78
**Renal disease**	79,369	28.51%	128,568	26.44%	4.64	8034	17.12%	27.39	51,341	19.16%	22.09
**MI**	39,359	14.14%	56,438	11.61%	7.57	4352	9.28%	15.17	26,012	9.71%	13.71
**Dyspepsia**	55,542	19.95%	90,915	18.70%	3.18	8326	17.75%	5.65	49,096	18.32%	4.15
**Non-stroke/SE PVD**	74,764	26.86%	128,098	26.34%	1.16	10,403	22.17%	10.91	62,895	23.47%	7.81
**History of stroke/SE**	43,187	15.51%	62,593	12.87%	7.58	5779	12.32%	9.25	28,595	10.67%	14.40
**TIA**	26,671	9.58%	54,832	11.28%	5.55	4237	9.03%	1.90	23,015	8.59%	3.46
**PAD**	72,575	26.07%	116,772	24.01%	4.75	9914	21.13%	11.66	58,789	21.94%	9.69
**CAD**	125,840	45.21%	202,793	41.70%	7.07	19,264	41.06%	8.38	106,088	39.59%	11.39
**Baseline Medication Use**											
**ACE/ARB**	127,797	45.91%	209,620	43.11%	5.64	22,060	47.02%	2.22	118,794	44.33%	3.18
**Amiodarone**	15,339	5.51%	30,664	6.31%	3.38	3437	7.33%	7.41	16,636	6.21%	2.97
**Beta-blockers**	168,481	60.52%	299,315	61.55%	2.11	29,395	62.65%	4.37	161,766	60.36%	0.33
**H2-RA**	19,696	7.08%	36,700	7.55%	1.81	3096	6.60%	1.89	18,342	6.84%	0.91
**Proton pump inhibitor**	81,777	29.38%	147,418	30.32%	2.05	13,470	28.71%	1.47	78,255	29.20%	0.39
**Statins**	154,028	55.33%	284,809	58.57%	6.55	26,654	56.81%	2.97	152,140	56.77%	2.90
**Anti-platelets**	40,204	14.44%	74,088	15.24%	2.23	6429	13.70%	2.13	37,481	13.99%	1.31
**NSAIDS**	51,427	18.47%	107,219	22.05%	8.90	10,260	21.87%	8.46	61,267	22.86%	10.85
**Dose**											
**Standard**			316,472	65.08%		37,763	80.48%		177,983	66.41%	
**Low**			112,522	23.14%		8566	18.26%		75,684	28.24%	
**Unknown**			57,263	11.78%		591	1.26%		14,324	5.34%	

Abbreviations: SD: standard deviation, STD: standardized mean difference (after multiplying by 100), LIS: low-income subsidy, SES: socio-economic status, CCI: Charlson comorbidity index, CHF: congestive heart failure, DM: diabetes mellitus, COPD: chronic obstructive pulmonary disease, MI: myocardial infarction, PVD: peripheral vascular disease, SE: systemic embolism, TIA: transient ischemic attack, PAD: peripheral arterial disease, CAD: coronary artery disease, ACE: angiotensin-converting enzyme, ARB: angiotensin receptor blockers, RA: receptor antagonist, NSAIDS: non-steroidal anti-inflammatory drug.

**Table 2:** Baseline demographic, socio-economic and clinical characteristics after IPTW weighting.

	Warfarin (Reference)		Apixaban Cohort			Dabigatran Cohort			Rivaroxaban Cohort		
	N/Mean	%/SD	N/Mean	%/SD	STD	N/Mean	%/SD	STD	N/Mean	%/SD	STD
**Sample Size**	278,372		486,257			46,920			267,991		
**Age**	77.68	7.64	77.71	7.55	0.33	77.97	7.59	3.77	77.84	7.51	2.12
**65–74**	108,514	38.98%	188,089	38.68%	0.62	17,183	36.62%	4.87	99,677	37.19%	3.68
**75–84**	111,375	40.01%	197,111	40.54%	1.07	19,561	41.69%	3.42	112,528	41.99%	4.03
**≥85**	58,482	21.01%	101,058	20.78%	0.56	10,176	21.69%	1.66	55,786	20.82%	0.47
**Gender**											
**Male**	143,021	51.38%	232,223	47.76%	7.25	23,566	50.23%	2.31	130,681	48.76%	5.23
**Female**	135,351	48.62%	254,034	52.24%	7.25	23,354	49.77%	2.31	137,310	51.24%	5.23
**Race**											
**White**	248,435	89.25%	435,383	89.54%	0.95	41,320	88.07%	3.72	238,211	88.89%	1.15
**Black**	15,842	5.69%	22,392	4.61%	4.92	2290	4.88%	3.62	12,718	4.75%	4.25
**Asian**	3581	1.29%	8690	1.79%	4.07	1123	2.39%	8.24	5287	1.97%	5.42
**Other**	10,514	3.78%	19,791	4.07%	1.51	2187	4.66%	4.40	11,775	4.39%	3.12
**U.S. Geographic Region**											
**Northeast**	50,905	18.29%	90,796	18.67%	0.99	8607	18.34%	0.15	50,193	18.73%	1.14
**Midwest**	73,096	26.26%	123,996	25.50%	1.73	11,069	23.59%	6.17	68,022	25.38%	2.00
**South**	104,805	37.65%	182,463	37.52%	0.26	18,455	39.33%	3.46	100,497	37.50%	0.31
**West**	48,825	17.54%	87,721	18.04%	1.31	8677	18.49%	2.48	48,535	18.11%	1.49
**Other**	741	0.27%	1280	0.26%	0.05	112	0.24%	0.56	744	0.28%	0.22
**Index Year**											
**2013**	32,673	11.74%	57,221	11.77%	0.09	5489	11.70%	0.12	31,572	11.78%	0.14
**2014**	37,141	13.34%	64,931	13.35%	0.03	6194	13.20%	0.42	35,803	13.36%	0.05
**2015**	38,018	13.66%	66,214	13.62%	0.12	6315	13.46%	0.58	36,554	13.64%	0.05
**2016**	41,625	14.95%	72,508	14.91%	0.12	6977	14.87%	0.24	39,814	14.86%	0.27
**2017**	41,045	14.74%	71,721	14.75%	0.01	6951	14.81%	0.20	39,651	14.80%	0.14
**2018**	44,508	15.99%	78,120	16.07%	0.21	7620	16.24%	0.69	42,990	16.04%	0.14
**2019**	43,361	15.58%	75,542	15.54%	0.11	7374	15.72%	0.39	41,606	15.53%	0.14
**Medicaid Dual Eligibility**	36,641	13.16%	67,633	13.91%	2.18	6734	14.35%	3.45	41,277	15.40%	6.40
**Part-D LIS**	50,393	18.10%	93,744	19.28%	3.02	9255	19.72%	4.14	55,148	20.58%	6.27
**SES Variable**											
**Low**	111,791	40.16%	193,877	39.87%	0.59	18,555	39.55%	1.25	108,330	40.42%	0.54
**Medium**	113,950	40.93%	197,743	40.67%	0.55	18,762	39.99%	1.93	108,149	40.36%	1.18
**High**	52,632	18.91%	94,637	19.46%	1.41	9603	20.47%	3.92	51,512	19.22%	0.80
**Baseline Comorbidity**											
**CCI**	5.50	2.38	5.44	2.34	2.71	5.49	2.35	0.70	5.45	2.34	2.40
**CHA_2_DS_2_-VASc Score**	4.47	1.70	4.45	1.70	1.12	4.50	1.72	2.02	4.47	1.71	0.25
**1**	6216	2.2%	10,153	2.1%	1.00	1022	2.2%	0.37	5577	2.1%	1.05
**2**	26,655	9.58%	49,172	10.11%	1.80	4501	9.59%	0.06	26,708	9.97%	1.32
**3**	52,967	19.03%	92,960	19.12%	0.23	8699	18.54%	1.25	50,438	18.82%	0.53
**4+**	192,534	69.16%	333,972	68.68%	1.04	32,699	69.69%	1.14	185,269	69.13%	0.07
**HAS-BLED Score**	2.62	1.22	2.61	1.15	0.33	2.64	1.17	1.61	2.61	1.15	0.32
**0**	9269	3.33%	12,641	2.60%	4.30	1233	2.63%	4.13	7199	2.69%	3.77
**1**	36,178	13.00%	53,352	10.97%	6.24	5412	11.53%	4.46	29,519	11.02%	6.10
**2**	93,053	33.43%	179,794	36.98%	7.43	16,526	35.22%	3.78	97,512	36.39%	6.21
**3+**	139,873	50.25%	240,470	49.45%	1.59	23,750	50.62%	0.74	133,761	49.91%	0.67
**Bleeding history**	53,652	19.27%	80,978	16.65%	6.83	8041	17.14%	5.54	44,932	16.77%	6.53
**Obesity**	63,903	22.96%	113,417	23.32%	0.87	11,089	23.63%	1.60	64,071	23.91%	2.25
**CHF**	83,527	30.01%	133,073	27.37%	5.84	12,821	27.33%	5.93	71,950	26.85%	7.00
**DM**	100,268	36.02%	167,980	34.55%	3.08	16,970	36.17%	0.31	96,236	35.91%	0.23
**Hypertension**	231,266	83.08%	414,315	85.20%	5.83	39,946	85.14%	5.63	229,382	85.59%	6.92
**COPD**	79,417	28.53%	139,573	28.70%	0.39	13,115	27.95%	1.28	77,132	28.78%	0.56
**Renal disease**	69,100	24.82%	120,015	24.68%	0.33	11,739	25.02%	0.45	66,530	24.83%	0.01
**MI**	36,548	13.13%	55,333	11.38%	5.34	4914	10.47%	8.24	28,819	10.75%	7.33
**Dyspepsia**	51,529	18.51%	92,371	19.00%	1.24	8512	18.14%	0.96	50,904	18.99%	1.24
**Non-stroke/SE PVD**	71,111	25.55%	122,597	25.21%	0.77	12,069	25.72%	0.41	69,832	26.06%	1.17
**History of stroke/SE**	38,715	13.91%	60,192	12.38%	4.53	6774	14.44%	1.52	32,170	12.00%	5.67
**TIA**	27,690	9.95%	49,647	10.21%	0.87	5286	11.27%	4.28	26,033	9.71%	0.78
**PAD**	66,614	23.93%	114,102	23.47%	1.09	11,277	24.04%	0.25	65,438	24.42%	1.14
**CAD**	118,333	42.51%	207,561	42.69%	0.36	19,877	42.36%	0.29	110,902	41.38%	2.28
**Baseline Medication Use**											
**ACE/ARB**	118,785	42.67%	219,108	45.06%	4.82	21,402	45.61%	5.93	120,243	44.87%	4.43
**Amiodarone**	14,525	5.22%	34,010	6.99%	7.42	3291	7.01%	7.50	17,000	6.34%	4.83
**Beta-blockers**	164,706	59.17%	308,115	63.36%	8.62	29,144	62.11%	6.03	163,400	60.97%	3.69
**H2-RA**	19,307	6.94%	35,529	7.31%	1.44	3409	7.26%	1.28	19,425	7.25%	1.22
**Proton pump inhibitor**	79,017	28.39%	148,681	30.58%	4.81	13,646	29.08%	1.54	80,300	29.96%	3.47
**Statins**	153,553	55.16%	285,758	58.77%	7.29	27,222	58.02%	5.77	154,454	57.63%	4.99
**Anti-platelets**	37,884	13.61%	76,188	15.67%	5.83	6844	14.59%	2.81	40,191	15.00%	3.97
**NSAIDS**	52,311	18.79%	106,896	21.98%	7.93	10,157	21.65%	7.11	60,853	22.71%	9.67
**Dose**											
**Standard**			336,915	69.29%		34,654	73.86%		167,082	62.35%	
**Low**			113,081	23.26%		9434	20.11%		82,557	30.81%	
**Unknown**			36,261	7.46%		2832	6.04%		18,352	6.85%	

Abbreviations: SD: Standard deviation, STD: standardized mean difference (after multiplying by 100), LIS: low-income subsidy, SES: socioeconomic status, CCI: Charlson comorbidity index, CHF: congestive heart failure, DM: diabetes mellitus, COPD: chronic obstructive pulmonary disease, MI: myocardial infarction, PVD: peripheral vascular disease, SE: systemic embolism, TIA: transient ischemic attack, PAD: peripheral arterial disease, CAD: coronary artery disease, ACE: angiotensin-converting enzyme, ARB: angiotensin receptor blockers, RA: receptor antagonist, NSAIDS: non-steroidal anti-inflammatory drug.

**Table 3:** Risk of stroke (HR, 95% CI) overall and by subgroups.

	Warfarin (Ref) vs. Apixaban		Apixaban vs. Dabigatran (Ref)		Apixaban vs. Rivaroxaban (Ref)		Warfarin (Ref) vs. Dabigatran		Warfarin (Ref) vs. Rivaroxaban		Rivaroxaban (Ref) vs. Dabigatran	
Overall												
**Stroke/SE**	0.69 (0.65,0.74)	**<0.0001**	0.88 (0.80,0.95)	**0.0029**	0.88 (0.84,0.92)	**<0.0001**	0.82 (0.69,0.98)	**0.0254**	0.77 (0.71,0.84)	**<0.0001**	1.01 (0.92,1.10)	0.8586
**Ischemic stroke**	0.75 (0.69,0.81)	**<0.0001**	0.82 (0.75,0.90)	**<0.0001**	0.93 (0.88,0.97)	**0.0012**	0.94 (0.78,1.13)	0.5025	0.78 (0.71,0.86)	**<0.0001**	1.14 (1.03,1.26)	**0.0102**
**Hemorrhagic stroke**	0.57 (0.49,0.67)	**<0.0001**	1.41 (1.09,1.85)	**0.0104**	0.76 (0.69,0.83)	**<0.0001**	0.53 (0.32,0.86)	**0.0105**	0.81 (0.67,0.98)	**0.0302**	0.53 (0.41,0.70)	**<0.0001**
**SE**	0.40 (0.29,0.55)	**<0.0001**	0.65 (0.43,0.98)	**0.0407**	0.65 (0.52,0.79)	**<0.0001**	0.27 (0.08,0.90)	**0.0334**	0.48 (0.32,0.73)	**0.0005**	0.98 (0.64,1.49)	0.9203
**65–74**												
**Stroke/SE**	0.73 (0.67,0.78)	**<0.0001**	0.92 (0.78,1.08)	0.3017	0.87 (0.80,0.94)	**0.0004**	0.79 (0.67,0.94)	**0.0067**	0.83 (0.77,0.91)	**<0.0001**	0.96 (0.81,1.13)	0.6096
**Ischemic stroke**	0.79 (0.72,0.87)	**<0.0001**	0.83 (0.69,0.98)	**0.033**	0.90 (0.82,0.98)	**0.0216**	0.97 (0.81,1.16)	0.7129	0.88 (0.79,0.97)	**0.0103**	1.10 (0.92,1.32)	0.2911
**Hemorrhagic stroke**	0.57 (0.48,0.67)	**<0.0001**	1.68 (1.02,2.78)	**0.0419**	0.82 (0.69,0.98)	**0.0312**	0.33 (0.20,0.55)	**<0.0001**	0.68 (0.57,0.82)	**<0.0001**	0.50 (0.30,0.82)	**0.0068**
**SE**	0.57 (0.39,0.84)	**0.0042**	0.97 (0.41,2.26)	0.9393	0.60 (0.41,0.87)	**0.0065**	0.60 (0.26,1.40)	0.2368	0.96 (0.66,1.41)	0.8440	0.62 (0.27,1.46)	0.2752
**75–84**												
**Stroke/SE**	0.67 (0.64,0.72)	**<0.0001**	0.92 (0.81,1.06)	0.2607	0.88 (0.83,0.94)	**0.0001**	0.73 (0.64,0.84)	**<0.0001**	0.76 (0.72,0.82)	**<0.0001**	0.96 (0.83,1.11)	0.5840
**Ischemic stroke**	0.75 (0.70,0.80)	**<0.0001**	0.89 (0.76,1.03)	0.1255	0.93 (0.87,1.00)	0.0649	0.85 (0.73,1.00)	**0.0442**	0.80 (0.74,0.87)	**<0.0001**	1.06 (0.91,1.24)	0.4435
**Hemorrhagic stroke**	0.49 (0.43,0.55)	**<0.0001**	1.57 (1.03,2.39)	**0.0356**	0.77 (0.67,0.89)	**0.0003**	0.31 (0.20,0.47)	**<0.0001**	0.64 (0.55,0.74)	**<0.0001**	0.48 (0.31,0.74)	**0.0008**
**SE**	0.50 (0.37,0.68)	**<0.0001**	0.41 (0.24,0.70)	**0.0011**	0.61 (0.44,0.83)	**0.0017**	1.22 (0.71,2.07)	0.4705	0.84 (0.62,1.15)	0.2782	1.43 (0.83,2.45)	0.1999
**85+**												
**Stroke/SE**	0.69 (0.66,0.72)	**<0.0001**	0.84 (0.71,1.00)	0.0517	0.90 (0.83,0.97)	**0.0074**	0.82 (0.74,0.90)	**<0.0001**	0.79 (0.75,0.83)	**<0.0001**	1.07 (0.89,1.28)	0.4666
**Ischemic stroke**	0.76 (0.73,0.80)	**<0.0001**	0.79 (0.66,0.96)	**0.0154**	0.95 (0.87,1.04)	0.2756	0.97 (0.87,1.08)	0.5527	0.81 (0.77,0.86)	**<0.0001**	1.21 (0.99,1.47)	0.0611
**Hemorrhagic stroke**	0.52 (0.48,0.57)	**<0.0001**	1.09 (0.67,1.78)	0.7364	0.71 (0.59,0.85)	**0.0002**	0.36 (0.27,0.48)	**<0.0001**	0.71 (0.64,0.79)	**<0.0001**	0.65 (0.39,1.07)	0.0872
**SE**	0.49 (0.40,0.60)	**<0.0001**	1.43 (0.43,4.78)	0.5597	0.81 (0.53,1.24)	0.3352	0.74 (0.48,1.15)	0.1830	0.80 (0.65,1.00)	**0.0471**	0.57 (0.17,1.94)	0.3653
**Male**												
**Stroke/SE**	0.74 (0.69,0.78)	**<0.0001**	0.80 (0.70,0.90)	**0.0003**	0.91 (0.85,0.97)	**0.0030**	0.93 (0.82,1.06)	0.2752	0.81 (0.76,0.87)	**<0.0001**	1.15 (1.02,1.31)	**0.0284**
**Ischemic stroke**	0.82 (0.77,0.88)	**<0.0001**	0.70 (0.62,0.80)	**<0.0001**	0.96 (0.89,1.04)	0.3257	1.18 (1.03,1.36)	**0.0165**	0.86 (0.79,0.93)	**0.0001**	1.39 (1.21,1.59)	**<0.0001**
**Hemorrhagic stroke**	0.52 (0.46,0.58)	**<0.0001**	1.92 (1.24,2.97)	**0.0034**	0.73 (0.63,0.83)	**<0.0001**	0.27 (0.17,0.41)	**<0.0001**	0.71 (0.62,0.81)	**<0.0001**	0.38 (0.25,0.59)	**<0.0001**
**SE**	0.69 (0.51,0.94)	**0.0201**	0.66 (0.36,1.21)	0.1830	0.99 (0.70,1.40)	0.9537	1.03 (0.56,1.90)	0.9138	0.70 (0.48,1.00)	**0.0484**	1.50 (0.80,2.80)	0.2100
**Female**												
**Stroke/SE**	0.73 (0.67,0.78)	**<0.0001**	0.98 (0.86,1.11)	0.7383	0.88 (0.83,0.93)	**<0.0001**	0.79 (0.67,0.94)	**0.0067**	0.83 (0.77,0.91)	**<0.0001**	0.90 (0.79,1.02)	0.0991
**Ischemic stroke**	0.79 (0.72,0.87)	**<0.0001**	0.97 (0.84,1.11)	0.6499	0.92 (0.87,0.98)	**0.0121**	0.97 (0.81,1.16)	0.7129	0.88 (0.79,0.97)	**0.0103**	0.96 (0.83,1.11)	0.5818
**Hemorrhagic stroke**	0.57 (0.48,0.67)	**<0.0001**	1.14 (0.82,1.59)	0.4398	0.80 (0.70,0.91)	**0.0006**	0.33 (0.20,0.55)	**<0.0001**	0.68 (0.57,0.82)	**<0.0001**	0.69 (0.49,0.97)	**0.0302**
**SE**	0.57 (0.39,0.84)	**0.0042**	0.68 (0.38,1.22)	0.1997	0.51 (0.39,0.66)	**<0.0001**	0.60 (0.26,1.40)	0.2368	0.96 (0.66,1.41)	0.8440	0.74 (0.41,1.32)	0.3039
**White**												
**Stroke/SE**	0.69 (0.66,0.72)	**<0.0001**	0.85 (0.77,0.93)	**0.0007**	0.88 (0.84,0.92)	**<0.0001**	0.82 (0.74,0.90)	**<0.0001**	0.79 (0.75,0.83)	**<0.0001**	1.04 (0.95,1.15)	0.4239
**Ischemic stroke**	0.76 (0.73,0.80)	**<0.0001**	0.79 (0.71,0.88)	**<0.0001**	0.94 (0.89,0.99)	**0.0186**	0.97 (0.87,1.08)	0.5527	0.81 (0.77,0.86)	**<0.0001**	1.20 (1.08,1.33)	**0.0007**
**Hemorrhagic stroke**	0.52 (0.48,0.57)	**<0.0001**	1.42 (1.07,1.89)	**0.0157**	0.73 (0.66,0.81)	**<0.0001**	0.36 (0.27,0.48)	**<0.0001**	0.71 (0.64,0.79)	**<0.0001**	0.51 (0.38,0.68)	**<0.0001**
**SE**	0.49 (0.40,0.60)	**<0.0001**	0.65 (0.42,1.02)	0.0614	0.61 (0.49,0.75)	**<0.0001**	0.74 (0.48,1.15)	0.1830	0.80 (0.65,1.00)	**0.0471**	0.92 (0.59,1.44)	0.7071
**Black**												
**Stroke/SE**	0.72 (0.63,0.83)	**<0.0001**	1.41 (0.93,2.14)	0.1050	0.90 (0.77,1.07)	0.2314	0.52 (0.34,0.79)	**0.0022**	0.81 (0.68,0.95)	**0.0109**	0.65 (0.42,1.00)	**0.0475**
**Ischemic stroke**	0.78 (0.67,0.91)	**0.0013**	1.68 (1.03,2.74)	**0.0390**	0.93 (0.77,1.11)	0.3925	0.47 (0.29,0.77)	**0.0028**	0.85 (0.71,1.02)	0.0782	0.56 (0.34,0.92)	**0.0232**
**Hemorrhagic stroke**	0.52 (0.36,0.75)	**0.0006**	0.62 (0.26,1.44)	0.2639	0.71 (0.45,1.11)	0.1286	0.84 (0.36,1.95)	0.6884	0.73 (0.47,1.12)	0.1476	1.16 (0.49,2.79)	0.7346
**SE**	0.54 (0.27,1.06)	0.0737	2.11 (0.14,31.25)	0.5861	1.45 (0.52,4.00)	0.4766	0.26 (0.02,3.70)	0.3162	0.38 (0.14,1.02)	0.0541	0.67 (0.04,10.84)	0.7794
**Asian**												
**Stroke/SE**	0.58 (0.44,0.76)	**<0.0001**	0.94 (0.55,1.60)	0.8126	0.72 (0.56,0.93)	**0.0104**	0.61 (0.35,1.05)	0.0751	0.81 (0.61,1.08)	0.1484	0.77 (0.45,1.32)	0.3436
**Ischemic stroke**	0.85 (0.61,1.20)	0.3655	0.78 (0.45,1.35)	0.3704	0.68 (0.52,0.90)	**0.0071**	1.08 (0.60,1.95)	0.8052	1.26 (0.89,1.79)	0.1984	0.88 (0.51,1.54)	0.6558
**Hemorrhagic stroke**	0.30 (0.18,0.50)	**<0.0001**	4.48 (0.36,55.56)	0.2449	0.99 (0.53,1.86)	0.9817	0.07 (0.01,0.82)	**0.0347**	0.31 (0.17,0.56)	**0.0001**	0.22 (0.02,2.82)	0.2446
**SE**	0.05 (0.01,0.45)	**0.007**	N/A	N/A	0.27 (0.02,3.17)	0.2989	N/A	N/A	0.17 (0.04,0.83)	**0.0281**	N/A	N/A
**Other**												
**Stroke/SE**	0.84 (0.69,1.02)	0.0732	0.95 (0.64,1.41)	0.8002	0.97 (0.81,1.18)	0.7842	0.89 (0.59,1.33)	0.5596	0.85 (0.69,1.06)	0.1588	1.03 (0.69,1.55)	0.8737
**Ischemic stroke**	0.81 (0.65,1.02)	0.0772	0.93 (0.59,1.47)	0.7564	0.88 (0.71,1.09)	0.2328	0.89 (0.55,1.42)	0.6208	0.91 (0.71,1.17)	0.4694	0.97 (0.61,1.55)	0.8993
**Hemorrhagic stroke**	0.89 (0.59,1.35)	0.5815	1.37 (0.51,3.66)	0.5336	1.34 (0.87,2.07)	0.1867	0.64 (0.23,1.77)	0.3871	0.68 (0.41,1.12)	0.1318	0.94 (0.34,2.63)	0.9079
**SE**	0.95 (0.38,2.38)	0.9179	0.47 (0.13,1.70)	0.2523	1.39 (0.54,3.60)	0.5013	2.06 (0.51,8.34)	0.3121	0.72 (0.24,2.17)	0.5579	2.77 (0.67,11.54)	0.1610
**Low SES**												
**Stroke/SE**	0.73 (0.69,0.77)	**<0.0001**	1.00 (0.87,1.15)	0.9833	0.88 (0.83,0.94)	**0.0001**	0.73 (0.63,0.84)	**<0.0001**	0.82 (0.77,0.88)	**<0.0001**	0.89 (0.77,1.02)	0.0994
**Ischemic stroke**	0.81 (0.76,0.87)	**<0.0001**	0.96 (0.83,1.12)	0.6254	0.93 (0.86,1.00)	**0.0445**	0.85 (0.73,0.99)	**0.0397**	0.88 (0.81,0.95)	**0.0009**	0.97 (0.83,1.14)	0.7408
**Hemorrhagic stroke**	0.52 (0.46,0.60)	**<0.0001**	1.39 (0.93,2.07)	0.1060	0.82 (0.71,0.96)	**0.0116**	0.37 (0.25,0.55)	**<0.0001**	0.63 (0.55,0.74)	**<0.0001**	0.59 (0.39,0.89)	**0.0109**
**SE**	0.44 (0.33,0.60)	**<0.0001**	0.68 (0.35,1.33)	0.2571	0.46 (0.34,0.62)	**<0.0001**	0.67 (0.34,1.29)	0.2263	0.98 (0.74,1.31)	0.8995	0.67 (0.34,1.30)	0.2326
**Medium SES**												
**Stroke/SE**	0.67 (0.63,0.71)	**<0.0001**	0.85 (0.73,0.98)	**0.0231**	0.85 (0.79,0.91)	**<0.0001**	0.80 (0.69,0.92)	**0.0023**	0.79 (0.74,0.85)	**<0.0001**	1.01 (0.87,1.17)	0.9407
**Ischemic stroke**	0.72 (0.67,0.78)	**<0.0001**	0.79 (0.68,0.93)	**0.0044**	0.89 (0.82,0.96)	**0.0037**	0.92 (0.78,1.08)	0.2798	0.81 (0.74,0.88)	**<0.0001**	1.13 (0.96,1.33)	0.1351
**Hemorrhagic stroke**	0.55 (0.48,0.63)	**<0.0001**	1.49 (0.96,2.34)	0.0788	0.72 (0.62,0.83)	**<0.0001**	0.36 (0.23,0.57)	**<0.0001**	0.77 (0.66,0.89)	**0.0007**	0.47 (0.30,0.74)	**0.0010**
**SE**	0.49 (0.36,0.66)	**<0.0001**	0.49 (0.27,0.88)	**0.0181**	0.74 (0.52,1.05)	0.0952	0.98 (0.54,1.78)	0.9547	0.66 (0.46,0.92)	**0.0158**	1.51 (0.81,2.79)	0.1930
**High SES**												
**Stroke/SE**	0.68 (0.62,0.74)	**<0.0001**	0.73 (0.61,0.87)	**0.0006**	0.95 (0.86,1.04)	0.2802	0.92 (0.77,1.12)	0.4069	0.71 (0.64,0.79)	**<0.0001**	1.32 (1.09,1.59)	**0.0043**
**Ischemic stroke**	0.74 (0.67,0.83)	**<0.0001**	0.65 (0.53,0.78)	**<0.0001**	1.00 (0.90,1.12)	0.9288	1.15 (0.93,1.41)	0.1894	0.74 (0.65,0.84)	**<0.0001**	1.57 (1.28,1.92)	**<0.0001**
**Hemorrhagic stroke**	0.50 (0.41,0.61)	**<0.0001**	1.37 (0.78,2.44)	0.2704	0.75 (0.61,0.91)	**0.0039**	0.36 (0.21,0.65)	**0.0005**	0.67 (0.54,0.83)	**0.0003**	0.55 (0.31,0.97)	**0.0401**
**SE**	0.60 (0.38,0.93)	**0.0239**	1.11 (0.38,3.23)	0.8489	1.25 (0.75,2.08)	0.3810	0.53 (0.18,1.57)	0.2491	0.47 (0.27,0.81)	**0.0073**	1.17 (0.38,3.40)	0.7881
**Dual**												
**Stroke/SE**	0.80 (0.73,0.87)	**<0.0001**	1.00 (0.82,1.22)	0.9611	1.00 (0.91,1.10)	0.9662	0.80 (0.65,0.98)	**0.0329**	0.79 (0.72,0.88)	**<0.0001**	1.01 (0.81,1.24)	0.9648
**Ischemic stroke**	0.92 (0.83,1.02)	0.1031	1.06 (0.76,1.32)	0.6333	1.03 (0.92,1.15)	0.5900	0.97 (0.77,1.22)	0.7989	0.88 (0.78,1.00)	**0.0454**	1.10 (0.87,1.39)	0.4120
**Hemorrhagic stroke**	0.55 (0.45,0.67)	**<0.0001**	0.59 (0.87,1.15)	0.1206	1.05 (0.83,1.34)	0.6820	0.32 (0.17,0.62)	**0.0007**	0.52 (0.40,0.66)	**<0.0001**	0.63 (0.32,1.23)	0.1715
**SE**	0.42 (0.28,0.64)	**<0.0001**	1.44 (0.26,3.78)	0.4549	0.52 (0.33,0.81)	**0.0040**	0.62 (0.24,1.60)	0.3209	0.83 (0.55,1.27)	0.3929	0.73 (0.28,1.91)	0.5210
**Non Dual**												
**Stroke/SE**	0.68 (0.65,0.71)	**<0.0001**	0.87 (0.79,0.96)	**0.0047**	0.85 (0.81,0.89)	**<0.0001**	0.79 (0.71,0.87)	**<0.0001**	0.81 (0.77,0.85)	**<0.0001**	0.98 (0.89,1.09)	0.7089
**Ischemic stroke**	0.75 (0.71,0.78)	**<0.0001**	0.81 (0.73,0.90)	**0.0001**	0.90 (0.85,0.95)	**<0.0001**	0.92 (0.83,1.03)	0.1488	0.83 (0.79,0.88)	**<0.0001**	1.11 (1.00,1.24)	0.0558
**Hemorrhagic stroke**	0.52 (0.47,0.57)	**<0.0001**	1.40 (1.04,1.87)	**0.0247**	0.71 (0.64,0.79)	**<0.0001**	0.37 (0.28,0.49)	**<0.0001**	0.73 (0.66,0.81)	**<0.0001**	0.50 (0.38,0.68)	**<0.0001**
**SE**	0.52 (0.42,0.65)	**<0.0001**	0.66 (0.41,1.04)	0.0703	0.68 (0.54,0.86)	**0.0010**	0.79 (0.50,1.25)	0.3135	0.77 (0.61,0.97)	**0.0257**	1.03 (0.65,1.65)	0.8889

Abbreviations: HR: hazard ratio, CI: confidence interval, SE: systemic embolism, SES: socioeconomic status.

**Table 4:** Risk (HR, 95% CI)) of MB overall and by subgroups.

	Apixaban vs. Warfarin (Ref)		Apixaban vs. Dabigatran (Ref)		Apixaban vs. Rivaroxaban (Ref)		Dabigatran vs. Warfarin (Ref)		Rivaroxaban vs. Warfarin (Ref)		Dabigatran vs. Rivaroxaban (Ref)	
Overall												
**MB**	0.59 (0.57,0.60)	**<0.0001**	0.76 (0.72,0.80)	**<0.0001**	0.60 (0.58,0.61)	**<0.0001**	0.77 (0.73,0.81)	**<0.0001**	0.99 (0.96,1.01)	0.3181	0.78 (0.73,0.82)	**<0.0001**
** GI bleeding**	0.61 (0.59,0.63)	**<0.0001**	0.63 (0.59,0.67)	**<0.0001**	0.52 (0.51,0.54)	**<0.0001**	0.96 (0.90,1.03)	0.2373	1.16 (1.12,1.19)	**<0.0001**	0.83 (0.78,0.88)	**<0.0001**
** Intracranial hemorrhage**	0.53 (0.51,0.56)	**<0.0001**	1.19 (1.03,1.39)	**0.0187**	0.86 (0.81,0.92)	**<0.0001**	0.44 (0.38,0.52)	**<0.0001**	0.62 (0.58,0.66)	**<0.0001**	0.72 (0.62,0.84)	**<0.0001**
**Other bleeding**	0.59 (0.56,0.63)	**<0.0001**	1.23 (1.02,1.47)	**0.0264**	0.68 (0.63,0.72)	**<0.0001**	0.48 (0.40,0.57)	**<0.0001**	0.88 (0.82,0.94)	**0.0003**	0.55 (0.46,0.66)	**<0.0001**
**65–74**												
**MB**	0.57 (0.54,0.60)	**<0.0001**	1.03 (0.92,1.15)	0.6346	0.63 (0.60,0.66)	**<0.0001**	0.55 (0.49,0.62)	**<0.0001**	0.90 (0.86,0.95)	**<0.0001**	0.61 (0.55,0.69)	**<0.0001**
**GI bleeding**	0.58 (0.55,0.62)	**<0.0001**	0.84 (0.74,0.96)	**0.0095**	0.57 (0.54,0.60)	**<0.0001**	0.69 (0.60,0.78)	**<0.0001**	1.02 (0.96,1.09)	0.4597	0.67 (0.59,0.76)	**<0.0001**
**Intracranial hemorrhage**	0.53 (0.47,0.59)	**<0.0001**	1.50 (1.09,2.07)	**0.0122**	0.90 (0.79,1.01)	0.0731	0.35 (0.25,0.48)	**<0.0001**	0.59 (0.52,0.67)	**<0.0001**	0.60 (0.43,0.83)	**0.0018**
**Other bleeding**	0.58 (0.52,0.66)	**<0.0001**	2.11 (1.42,3.12)	**0.0002**	0.70 (0.62,0.79)	**<0.0001**	0.28 (0.19,0.41)	**<0.0001**	0.83 (0.73,0.93)	**0.0025**	0.34 (0.23,0.50)	**<0.0001**
**75–84**												
**MB**	0.58 (0.56,0.60)	**<0.0001**	0.77 (0.71,0.84)	**<0.0001**	0.61 (0.58,0.63)	**<0.0001**	0.75 (0.69,0.82)	**<0.0001**	0.96 (0.93,1.00)	0.0582	0.78 (0.71,0.85)	**<0.0001**
**GI bleeding**	0.61 (0.58,0.64)	**<0.0001**	0.65 (0.59,0.71)	**<0.0001**	0.53 (0.51,0.56)	**<0.0001**	0.94 (0.85,1.03)	0.1868	1.14 (1.09,1.20)	**<0.0001**	0.82 (0.74,0.90)	**<0.0001**
**Intracranial hemorrhage**	0.51 (0.47,0.55)	**<0.0001**	1.19 (0.95,1.49)	0.1383	0.90 (0.82,0.99)	**0.0227**	0.43 (0.34,0.54)	**<0.0001**	0.57 (0.52,0.62)	**<0.0001**	0.75 (0.60,0.95)	**0.0161**
**Other bleeding**	0.59 (0.54,0.65)	**<0.0001**	1.17 (0.89,1.54)	0.2624	0.68 (0.62,0.76)	**<0.0001**	0.50 (0.38,0.66)	**<0.0001**	0.87 (0.78,0.97)	**0.0100**	0.58 (0.44,0.76)	**<0.0001**
**85+**												
**MB**	0.63 (0.60,0.66)	**<0.0001**	0.63 (0.56,0.70)	**<0.0001**	0.56 (0.53,0.59)	**<0.0001**	1.01 (0.90,1.12)	0.8864	1.13 (1.08,1.19)	**<0.0001**	0.89 (0.79,0.99)	**0.0302**
**GI bleeding**	0.64 (0.61,0.68)	**<0.0001**	0.50 (0.44,0.57)	**<0.0001**	0.48 (0.45,0.51)	**<0.0001**	1.28 (1.13,1.45)	**0.0001**	1.34 (1.26,1.43)	**<0.0001**	0.95 (0.84,1.08)	0.4499
**Intracranial hemorrhage**	0.60 (0.54,0.65)	**<0.0001**	1.10 (0.83,1.46)	0.4934	0.81 (0.73,0.91)	**0.0002**	0.54 (0.41,0.72)	**<0.0001**	0.74 (0.66,0.82)	**<0.0001**	0.73 (0.55,0.98)	**0.0357**
**Other bleeding**	0.65 (0.57,0.74)	**<0.0001**	0.92 (0.65,1.31)	0.6544	0.65 (0.57,0.75)	**<0.0001**	0.69 (0.49,0.99)	**0.0427**	0.99 (0.86,1.15)	0.9019	0.70 (0.49,1.01)	0.0537
**Male**												
**MB**	0.58 (0.56,0.60)	**<0.0001**	0.82 (0.76,0.89)	**<0.0001**	0.61 (0.59,0.63)	**<0.0001**	0.70 (0.65,0.76)	**<0.0001**	0.95 (0.92,0.99)	**0.013**	0.74 (0.68,0.80)	**<0.0001**
**GI bleeding**	0.59 (0.56,0.62)	**<0.0001**	0.67 (0.60,0.73)	**<0.0001**	0.53 (0.50,0.55)	**<0.0001**	0.88 (0.80,0.97)	**0.0103**	1.12 (1.07,1.17)	**<0.0001**	0.79 (0.71,0.87)	**<0.0001**
**Intracranial hemorrhage**	0.54 (0.51,0.59)	**<0.0001**	1.57 (1.25,2.00)	**0.0002**	0.92 (0.84,1.00)	0.0536	0.35 (0.27,0.44)	**<0.0001**	0.59 (0.54,0.65)	**<0.0001**	0.58 (0.46,0.74)	**<0.0001**
**Other bleeding**	0.60 (0.55,0.65)	**<0.0001**	1.03 (0.82,1.29)	0.8230	0.67 (0.61,0.74)	**<0.0001**	0.58 (0.46,0.72)	**<0.0001**	0.88 (0.80,0.97)	**0.0123**	0.65 (0.52,0.82)	**0.0002**
**Female**												
**MB**	0.60 (0.58,0.62)	**<0.0001**	0.72 (0.66,0.77)	**<0.0001**	0.59 (0.57,0.61)	**<0.0001**	0.84 (0.77,0.90)	**<0.0001**	1.03 (0.99,1.07)	0.1536	0.81 (0.75,0.87)	**<0.0001**
**GI bleeding**	0.62 (0.60,0.65)	**<0.0001**	0.60 (0.55,0.65)	**<0.0001**	0.52 (0.50,0.55)	**<0.0001**	1.04 (0.95,1.13)	0.4362	1.19 (1.14,1.25)	**<0.0001**	0.86 (0.79,0.94)	**0.0011**
**Intracranial hemorrhage**	0.54 (0.50,0.58)	**<0.0001**	0.98 (0.81,1.19)	0.8422	0.83 (0.76,0.90)	**<0.0001**	0.54 (0.45,0.66)	**<0.0001**	0.65 (0.60,0.71)	**<0.0001**	0.83 (0.68,1.02)	0.0758
**Other bleeding**	0.61 (0.56,0.67)	**<0.0001**	1.65 (1.21,2.25)	**0.0016**	0.69 (0.62,0.76)	**<0.0001**	0.37 (0.27,0.50)	**<0.0001**	0.89 (0.80,0.99)	**0.0313**	0.41 (0.30,0.57)	**<0.0001**
**White**												
**MB**	0.60 (0.58,0.62)	**<0.0001**	0.74 (0.70,0.79)	**<0.0001**	0.59 (0.58,0.61)	**<0.0001**	0.80 (0.75,0.85)	**<0.0001**	1.02 (0.99,1.04)	0.2856	0.79 (0.74,0.83)	**<0.0001**
**GI bleeding**	0.62 (0.60,0.65)	**<0.0001**	0.61 (0.57,0.66)	**<0.0001**	0.52 (0.51,0.54)	**<0.0001**	1.01 (0.95,1.09)	0.7226	1.20 (1.16,1.24)	**<0.0001**	0.84 (0.79,0.90)	**<0.0001**
**Intracranial hemorrhage**	0.54 (0.51,0.57)	**<0.0001**	1.20 (1.02,1.41)	**0.0250**	0.85 (0.80,0.90)	**<0.0001**	0.45 (0.38,0.52)	**<0.0001**	0.63 (0.59,0.68)	**<0.0001**	0.70 (0.60,0.83)	**<0.0001**
**Other bleeding**	0.60 (0.56,0.64)	**<0.0001**	1.20 (0.99,1.45)	0.0599	0.67 (0.63,0.72)	**<0.0001**	0.49 (0.41,0.60)	**<0.0001**	0.89 (0.83,0.96)	**0.0031**	0.56 (0.46,0.67)	**<0.0001**
**Black**												
**MB**	0.59 (0.54,0.65)	**<0.0001**	0.88 (0.69,1.13)	0.3120	0.56 (0.50,0.62)	**<0.0001**	0.67 (0.53,0.86)	**0.0016**	1.06 (0.96,1.18)	0.2534	0.63 (0.49,0.81)	**0.0003**
**GI bleeding**	0.61 (0.55,0.69)	**<0.0001**	0.79 (0.60,1.05)	0.1017	0.52 (0.46,0.58)	**<0.0001**	0.77 (0.58,1.02)	0.0633	1.18 (1.05,1.33)	**0.0055**	0.65 (0.49,0.86)	**0.0024**
**Intracranial hemorrhage**	0.50 (0.39,0.64)	**<0.0001**	0.78 (0.41,1.45)	0.4279	0.83 (0.60,1.13)	0.2316	0.66 (0.35,1.22)	0.1843	0.61 (0.45,0.82)	**0.0013**	1.08 (0.56,2.07)	0.8219
**Other bleeding**	0.60 (0.46,0.78)	**0.0001**	3.11 (0.91,10.53)	0.0696	0.61 (0.46,0.82)	**0.0009**	0.19 (0.06,0.65)	**0.0081**	0.98 (0.73,1.30)	0.8592	0.20 (0.06,0.67)	**0.0094**
**Asian**												
**MB**	0.46 (0.38,0.56)	**<0.0001**	0.73 (0.50,1.07)	0.1112	0.77 (0.63,0.94)	**0.0107**	0.64 (0.43,0.94)	**0.0221**	0.61 (0.49,0.75)	**<0.0001**	1.03 (0.70,1.52)	0.8690
**GI bleeding**	0.37 (0.28,0.49)	**<0.0001**	0.50 (0.31,0.81)	**0.0048**	0.54 (0.41,0.72)	**<0.0001**	0.75 (0.47,1.21)	0.2419	0.68 (0.52,0.90)	**0.0065**	1.08 (0.67,1.74)	0.7517
**Intracranial hemorrhage**	0.55 (0.40,0.76)	**0.0003**	1.33 (0.63,2.82)	0.4594	1.36 (0.94,1.96)	0.1016	0.42 (0.20,0.91)	**0.0282**	0.41 (0.27,0.61)	**<0.0001**	1.01 (0.46,2.22)	0.9854
**Other bleeding**	0.67 (0.34,1.32)	0.2525	0.85 (0.25,2.89)	0.7924	0.72 (0.40,1.32)	0.2944	0.81 (0.22,2.89)	0.7396	0.98 (0.49,1.99)	0.9593	0.83 (0.24,2.88)	0.7696
**Other**												
**MB**	0.56 (0.49,0.64)	**<0.0001**	1.09 (0.79,1.51)	0.5892	0.77 (0.67,0.88)	**0.0001**	0.51 (0.37,0.71)	**<0.0001**	0.74 (0.64,0.85)	**<0.0001**	0.70 (0.50,0.97)	**0.0298**
**GI bleeding**	0.50 (0.42,0.59)	**<0.0001**	0.88 (0.60,1.28)	0.5001	0.63 (0.54,0.75)	**<0.0001**	0.57 (0.39,0.83)	**0.0036**	0.79 (0.66,0.94)	**0.0073**	0.72 (0.49,1.05)	0.0890
**Intracranial hemorrhage**	0.64 (0.49,0.83)	**0.0007**	1.77 (0.81,3.86)	0.1514	1.12 (0.84,1.49)	0.4474	0.36 (0.16,0.78)	**0.0102**	0.58 (0.43,0.79)	**0.0006**	0.61 (0.28,1.36)	0.2303
**Other bleeding**	0.85 (0.56,1.28)	0.4236	1.58 (0.56,4.48)	0.3922	1.06 (0.70,1.59)	0.7817	0.54 (0.19,1.58)	0.2598	0.79 (0.49,1.27)	0.3273	0.69 (0.24,2.00)	0.4908
**Low SES**												
**MB**	0.60 (0.57,0.62)	**<0.0001**	0.76 (0.70,0.83)	**<0.0001**	0.61 (0.59,0.63)	**<0.0001**	0.78 (0.72,0.85)	**<0.0001**	0.98 (0.94,1.02)	0.3219	0.79 (0.73,0.87)	**<0.0001**
**GI bleeding**	0.62 (0.59,0.65)	**<0.0001**	0.65 (0.59,0.72)	**<0.0001**	0.54 (0.52,0.57)	**<0.0001**	0.94 (0.85,1.04)	0.2065	1.14 (1.09,1.19)	**<0.0001**	0.82 (0.75,0.91)	**<0.0001**
**Intracranial hemorrhage**	0.53 (0.49,0.58)	**<0.0001**	1.07 (0.86,1.34)	0.5523	0.90 (0.81,0.99)	**0.0321**	0.50 (0.40,0.62)	**<0.0001**	0.59 (0.54,0.65)	**<0.0001**	0.84 (0.67,1.06)	0.1366
**Other bleeding**	0.61 (0.55,0.67)	**<0.0001**	1.23 (0.93,1.63)	0.1536	0.70 (0.63,0.78)	**<0.0001**	0.49 (0.37,0.64)	**<0.0001**	0.87 (0.78,0.97)	**0.0097**	0.56 (0.42,0.75)	**<0.0001**
**Medium SES**												
**MB**	0.60 (0.58,0.63)	**<0.0001**	0.77 (0.70,0.84)	**<0.0001**	0.59 (0.56,0.61)	**<0.0001**	0.78 (0.71,0.85)	**<0.0001**	1.03 (0.99,1.07)	0.1760	0.75 (0.68,0.82)	**<0.0001**
**GI bleeding**	0.60 (0.57,0.64)	**<0.0001**	0.62 (0.56,0.69)	**<0.0001**	0.51 (0.48,0.53)	**<0.0001**	0.97 (0.87,1.08)	0.5205	1.19 (1.13,1.25)	**<0.0001**	0.81 (0.73,0.90)	**<0.0001**
**Intracranial hemorrhage**	0.56 (0.51,0.60)	**<0.0001**	1.34 (1.04,1.73)	**0.0227**	0.86 (0.78,0.95)	**0.0019**	0.41 (0.32,0.53)	**<0.0001**	0.65 (0.59,0.72)	**<0.0001**	0.63 (0.49,0.82)	**0.0005**
**Other bleeding**	0.67 (0.61,0.75)	**<0.0001**	1.17 (0.88,1.55)	0.2889	0.68 (0.61,0.75)	**<0.0001**	0.58 (0.43,0.77)	**0.0001**	0.99 (0.89,1.11)	0.8913	0.58 (0.44,0.78)	**0.0002**
**High SES**												
**MB**	0.56 (0.52,0.59)	**<0.0001**	0.75 (0.66,0.85)	**<0.0001**	0.59 (0.55,0.62)	**<0.0001**	0.75 (0.65,0.85)	**<0.0001**	0.95 (0.89,1.02)	0.1447	0.78 (0.69,0.89)	**0.0002**
**GI bleeding**	0.62 (0.57,0.67)	**<0.0001**	0.59 (0.51,0.68)	**<0.0001**	0.52 (0.49,0.56)	**<0.0001**	1.05 (0.90,1.22)	0.5539	1.18 (1.09,1.29)	**<0.0001**	0.88 (0.76,1.02)	0.0929
**Intracranial hemorrhage**	0.50 (0.44,0.57)	**<0.0001**	1.21 (0.87,1.68)	0.2473	0.81 (0.71,0.92)	**0.0009**	0.41 (0.30,0.57)	**<0.0001**	0.62 (0.54,0.71)	**<0.0001**	0.67 (0.48,0.93)	**0.0180**
**Other bleeding**	0.46 (0.39,0.53)	**<0.0001**	1.45 (0.91,2.29)	0.1154	0.61 (0.52,0.71)	**<0.0001**	0.31 (0.20,0.50)	**<0.0001**	0.75 (0.64,0.88)	**0.0005**	0.42 (0.26,0.66)	**0.0002**
**Dual**												
**MB**	0.59 (0.56,0.63)	**<0.0001**	0.83 (0.72,0.95)	**0.0073**	0.65 (0.61,0.69)	**<0.0001**	0.72 (0.62,0.82)	**<0.0001**	0.91 (0.86,0.97)	**0.0043**	0.78 (0.68,0.90)	**0.0005**
**GI bleeding**	0.57 (0.53,0.62)	**<0.0001**	0.68 (0.58,0.80)	**<0.0001**	0.56 (0.52,0.60)	**<0.0001**	0.84 (0.72,0.98)	**0.0295**	1.03 (0.96,1.11)	0.4469	0.82 (0.70,0.95)	**0.0102**
**Intracranial hemorrhage**	0.59 (0.51,0.67)	**<0.0001**	1.19 (0.83,1.71)	0.3396	1.07 (0.92,1.26)	0.3823	0.49 (0.34,0.70)	**0.0001**	0.55 (0.46,0.64)	**<0.0001**	0.89 (0.62,1.30)	0.5579
**Other bleeding**	0.72 (0.61,0.84)	**<0.0001**	1.80 (1.08,3.00)	**0.0247**	0.82 (0.69,0.97)	**0.0242**	0.40 (0.24,0.66)	**0.0004**	0.88 (0.74,1.06)	0.1795	0.45 (0.27,0.76)	**0.0026**
**Non Dual**												
**MB**	0.59 (0.57,0.60)	**<0.0001**	0.74 (0.69,0.79)	**<0.0001**	0.58 (0.57,0.60)	**<0.0001**	0.79 (0.74,0.84)	**<0.0001**	1.01 (0.99,1.04)	0.3478	0.78 (0.73,0.83)	**<0.0001**
**GI bleeding**	0.61 (0.59,0.64)	**<0.0001**	0.61 (0.57,0.65)	**<0.0001**	0.51 (0.50,0.53)	**<0.0001**	1.00 (0.94,1.08)	0.9104	1.20 (1.16,1.24)	**<0.0001**	0.84 (0.78,0.90)	**<0.0001**
**Intracranial hemorrhage**	0.53 (0.50,0.56)	**<0.0001**	1.22 (1.03,1.44)	**0.0195**	0.83 (0.78,0.89)	**<0.0001**	0.43 (0.37,0.51)	**<0.0001**	0.64 (0.60,0.68)	**<0.0001**	0.68 (0.57,0.80)	**<0.0001**
**Other bleeding**	0.58 (0.54,0.62)	**<0.0001**	1.14 (0.94,1.38)	0.1982	0.65 (0.60,0.70)	**<0.0001**	0.50 (0.41,0.61)	**<0.0001**	0.89 (0.83,0.97)	**0.0042**	0.57 (0.46,0.69)	**<0.0001**

Abbreviations: HR: hazard ratio, CI: confidence interval, SES: socioeconomic status.

## Data Availability

Restrictions apply to the availability of these data. The datasets generated and/or analyzed for the current study are not publicly available due to the confidential and proprietary nature of the datasets.

## Supplementary Materials

The following supporting information can be downloaded at: https://www.mdpi.com/article/10.3390/jcm14093252/s1, Figure S1: Comparison between apixaban vs. warfarin on the risk of stroke/SE among the overall population and by demographic and socioeconomic status subgroups; Figure S2: Comparison between apixaban vs. warfarin on the risk of major bleeding among the overall population and by demographic and socioeconomic status subgroups; Figure S3: Comparison between apixaban vs. rivaroxaban on the risk of stroke/se among the overall population and by demographic and socioeconomic status subgroups; Figure S4: Comparison between apixaban vs. rivaroxaban on the risk of major bleeding among the overall population and by demographic and socioeconomic status subgroups; Figure S5: Comparison between apixaban vs. dabigatran on the risk of stroke/se among the overall population and by demographic and socioeconomic status subgroups; Figure S6: Comparison between apixaban vs. dabigatran on the risk of major bleeding among the overall population and by demographic and socioeconomic status subgroups; Figure S7: Comparison between dabigatran vs. rivaroxaban on the risk of stroke/se among the overall population and by demographic and socioeconomic status subgroups; Figure S8: Comparison between dabigatran vs. rivaroxaban on the risk of major bleeding among the overall population and by demographic and socioeconomic status subgroups; Figure S9: Comparison between rivaroxaban vs. warfarin on the risk of stroke/se among the overall population and by demographic and socioeconomic status subgroups; Figure S10: Comparison between rivaroxaban vs. warfarin on the risk of major bleeding among the overall population and by demographic and socioeconomic status subgroups; Figure S11: Comparison between dabigatran vs. warfarin on the risk of stroke/se among the overall population and by demographic and socioeconomic status subgroups; Figure S12: Comparison between dabigatran vs. warfarin on the risk of major bleeding among the overall population and by demographic and socioeconomic status subgroups; Table S1: ICD codes for atrial fibrillation; Table S2: ICD codes for stroke event; Table S3: ICD codes for bleeding events.

## Abbreviations

The following abbreviations are used in this manuscript:

ACE	angiotensin-converting enzyme
AF	atrial fibrillation
ARB	angiotensin receptor blocker
CAD	coronary artery disease
CCI	Deyo–Charlson comorbidity index
CHF	congestive heart failure
CI	confidence interval
CMS	US Centers for Medicare and Medicaid Services
COPD	chronic obstructive pulmonary disease
DM	diabetes mellitus
FFS	fee-for-service
FPL	federal poverty level
DOAC	direct-acting oral anticoagulants
HIPAA	Health Insurance Portability and Accountability Act
HR	hazard ratio
ICD-9-CM	International Classification of Diseases, Ninth Revision, Clinical Modification
ICD-10-CM	International Classification of Diseases, Tenth Revision, Clinical Modification
INR	international normalized ratio
IPTW	inverse probability treatment weighting
IRB	institutional review board
LIS	low-income subsidy
MB	major bleeding
MI	myocardial infarction
NSAID	nonsteroidal anti-inflammatory drugs
NVAF	nonvalvular atrial fibrillation
OAC	oral anticoagulant
PAD	peripheral arterial disease
PVD	peripheral vascular disease
RA	receptor antagonist
SD	standard deviation
SE	systemic embolism
SES	socio-economic status
STD	standardized mean difference
TIA	transient ischemic attack
US	United States
VKA	vitamin K antagonist
