# Supplementary material for: Demographic and Socio-Economic Disparities in the Outcomes Among Patients with NVAF Treated with Oral Anticoagulants: A Real-World Evaluation of Medicare Beneficiaries
Source: J Clin Med. 2025 May 7;14(9):3252. doi: 10.3390/jcm14093252 (PMC12072770; doi:10.3390/jcm14093252)
Supplement: Supplementary file 1 [file jcm-14-03252-s001.zip › jcm-3582385-supplementary.pdf]

ONLINE SUPPLEMENT

Demographic and Socio-Economic Disparities in the Outcomes Among Patients with NVAF Treated with Oral Anticoagulants: A Real-World Evaluation of Medicare Beneficiaries

Supplemental Figures List:

Figure S1: Comparison between Apixaban vs. Warfarin on the Risk of stroke/SE among the Overall Population and by Demographic and Socioeconomic Status Subgroups 2
Figure S2: Comparison between Apixaban vs. Warfarin on the Risk of Major Bleeding among the Overall Population and by Demographic and Socioeconomic Status Subgroups 3
Figure S3: Comparison between Apixaban vs. Rivaroxaban on the Risk of stroke/SE among the Overall Population and by Demographic and Socioeconomic Status Subgroups 4
Figure S4: Comparison between Apixaban vs. Rivaroxaban on the Risk of Major Bleeding among the Overall Population and by Demographic and Socioeconomic Status Subgroups 5
Figure S5: Comparison between Apixaban vs. Dabigatran on the Risk of stroke/SE among the Overall Population and by Demographic and Socioeconomic Status Subgroups 6
Figure S6: Comparison between Apixaban vs. Dabigatran on the Risk of Major Bleeding among the Overall Population and by Demographic and Socioeconomic Status Subgroups 7
Figure S7: Comparison between Dabigatran vs. Rivaroxaban on the Risk of stroke/SE among the Overall Population and by Demographic and Socioeconomic Status Subgroups 8
Figure S8: Comparison between Dabigatran vs. Rivaroxaban on the Risk of Major Bleeding among the Overall Population and by Demographic and Socioeconomic Status Subgroups 9
Figure S9: Comparison between Rivaroxaban vs. Warfarin on the Risk of stroke/SE among the Overall Population and by Demographic and Socioeconomic Status Subgroups 10
Figure S10: Comparison between Rivaroxaban vs. Warfarin on the Risk of Major Bleeding among the Overall Population and by Demographic and Socioeconomic Status Subgroups 11
Figure S11: Comparison between Dabigatran vs. Warfarin on the Risk of stroke/SE among the Overall Population and by Demographic and Socioeconomic Status Subgroups 12
Figure S12: Comparison between Dabigatran vs. Warfarin on the Risk of Major Bleeding among the Overall Population and by Demographic and Socioeconomic Status Subgroups 13

Supplemental Table List:

Table S1: ICD codes for Atrial fibrillation 14
Table S2: ICD codes for Stroke Event 14
Table S3: ICD codes for Bleeding Events 20

Figure S1: Comparison between Apixaban vs. Warfarin on the Risk of stroke/SE among the Overall Population and by Demographic and Socioeconomic Status Subgroups

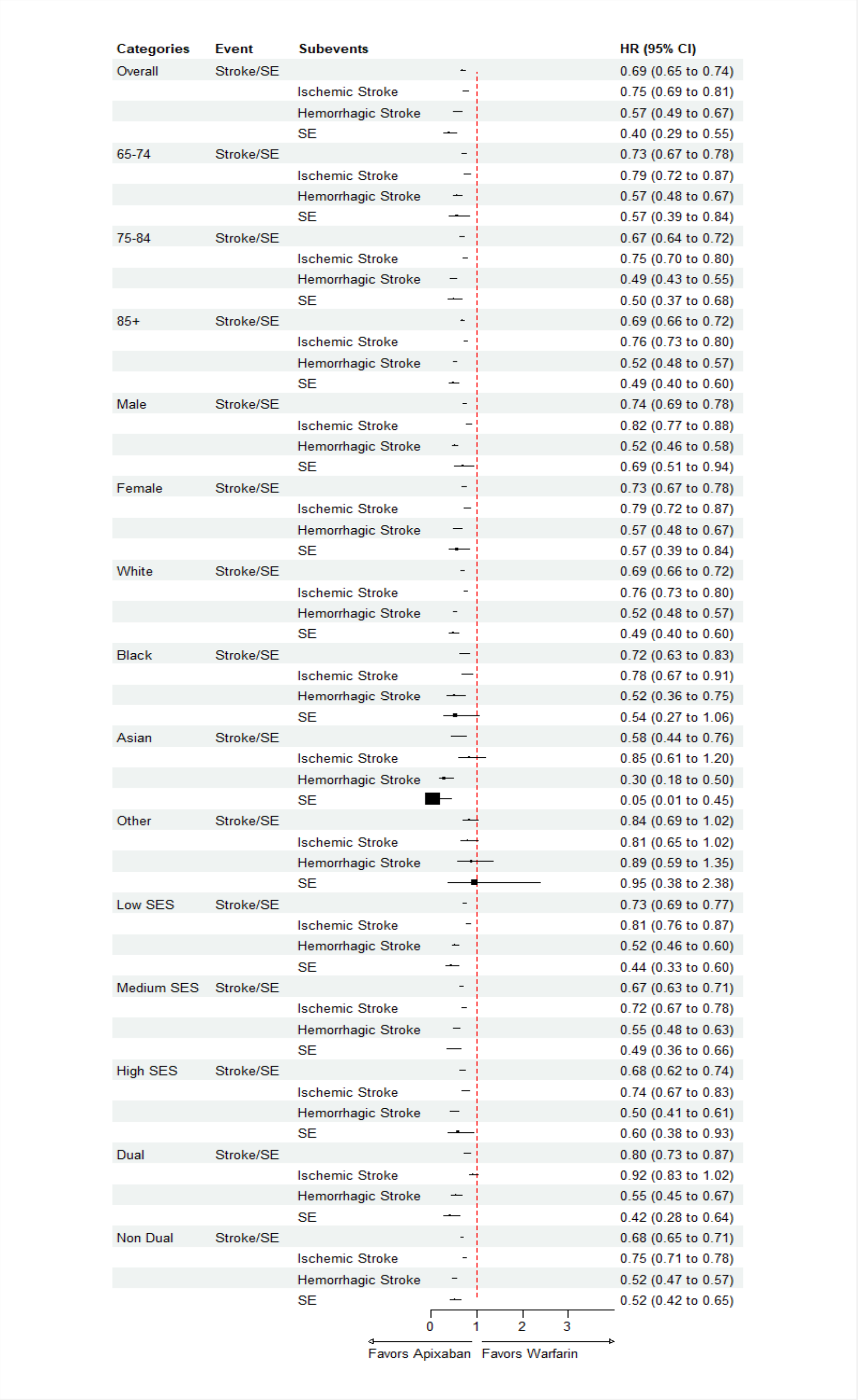

SE: Systemic Embolism; SES: Socioeconomic Status

Figure S2: Comparison between Apixaban vs. Warfarin on the Risk of Major Bleeding among the Overall Population and by Demographic and Socioeconomic Status Subgroups

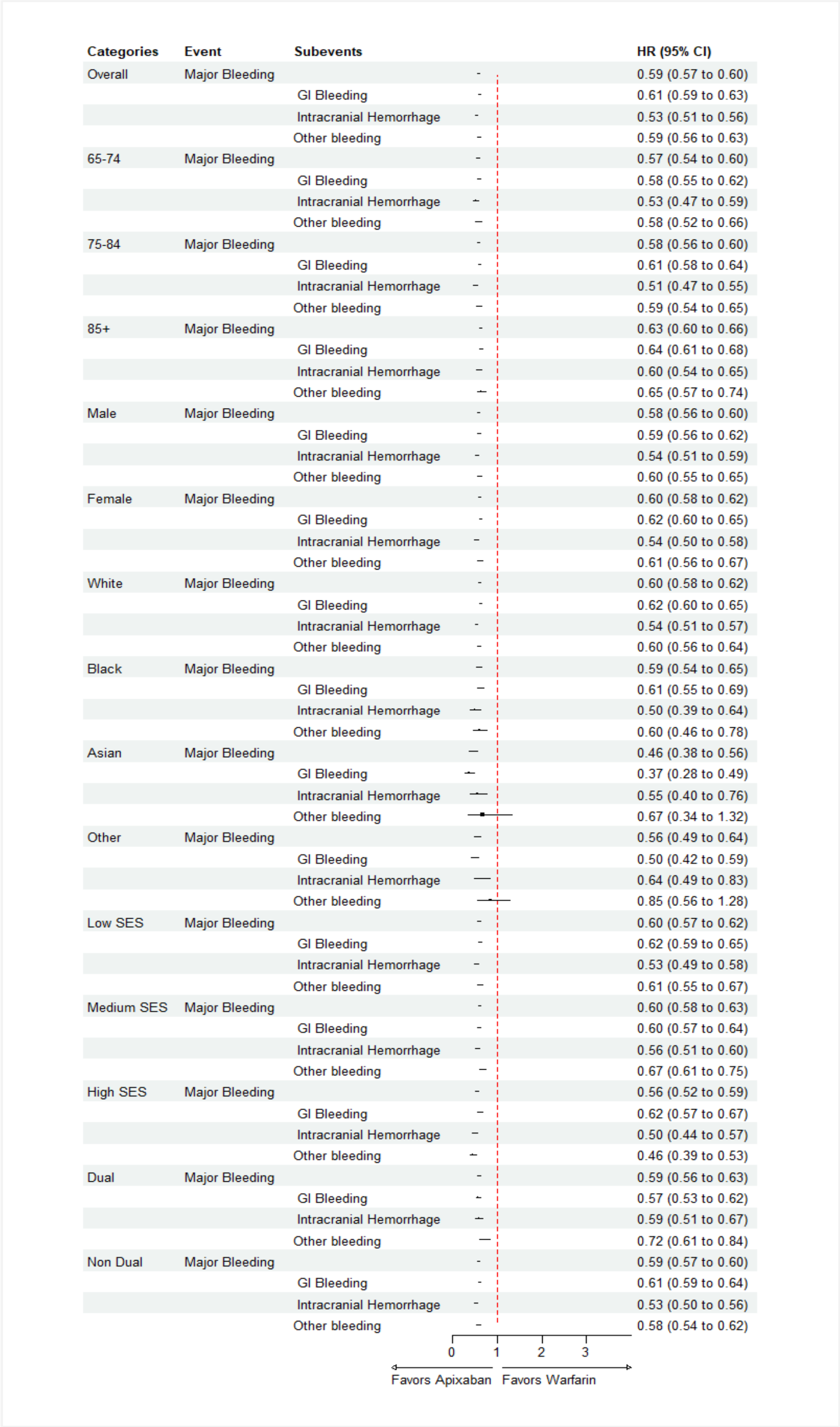

GI: Gastrointestinal; SES: Socioeconomic Status

Figure S3: Comparison between Apixaban vs. Rivaroxaban on the Risk of stroke/SE among the Overall Population and by Demographic and Socioeconomic Status Subgroups

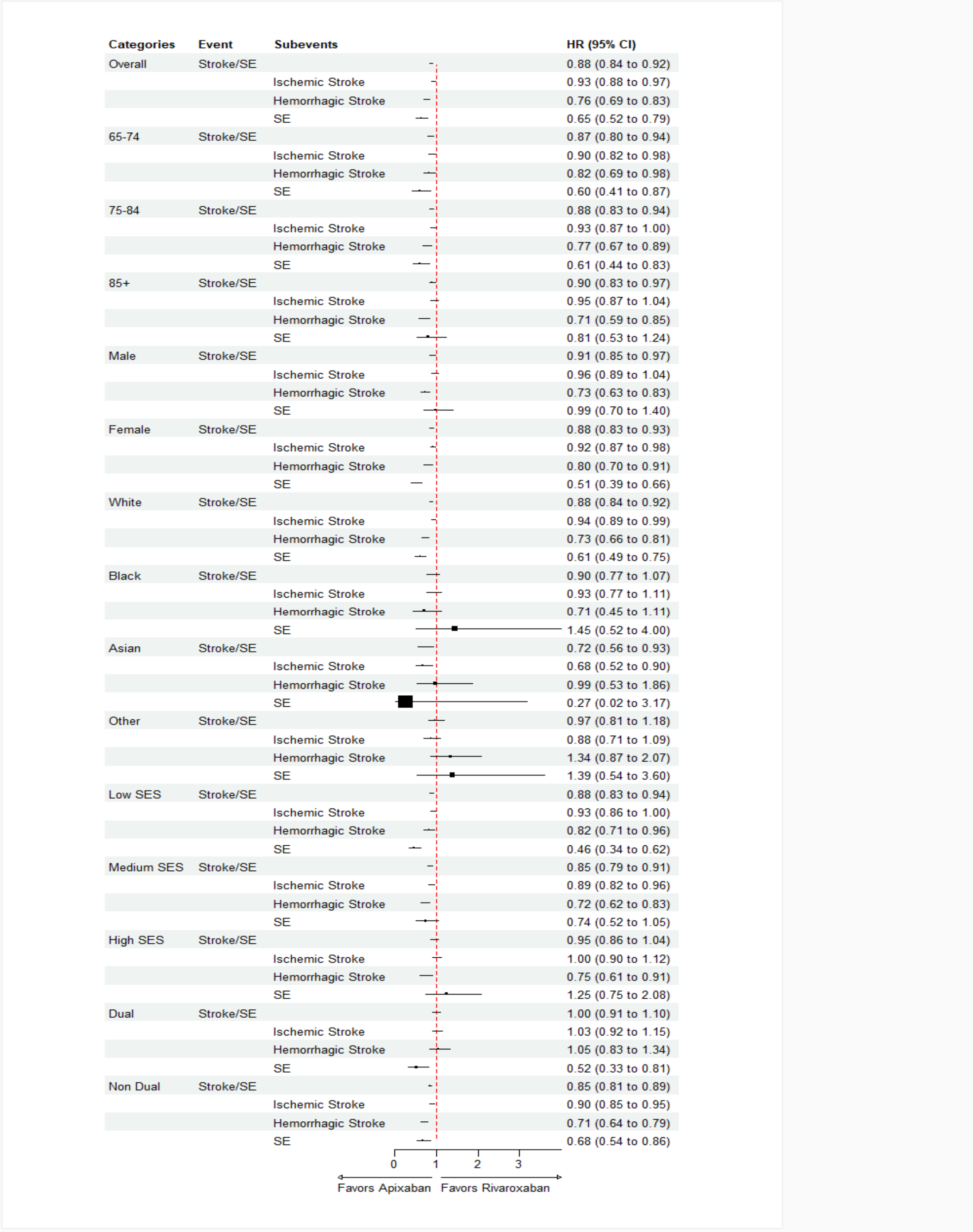

SE: Systemic Embolism; SES: Socioeconomic Status

Figure S4: Comparison between Apixaban vs. Rivaroxaban on the Risk of Major Bleeding among the Overall Population and by Demographic and Socioeconomic Status Subgroups

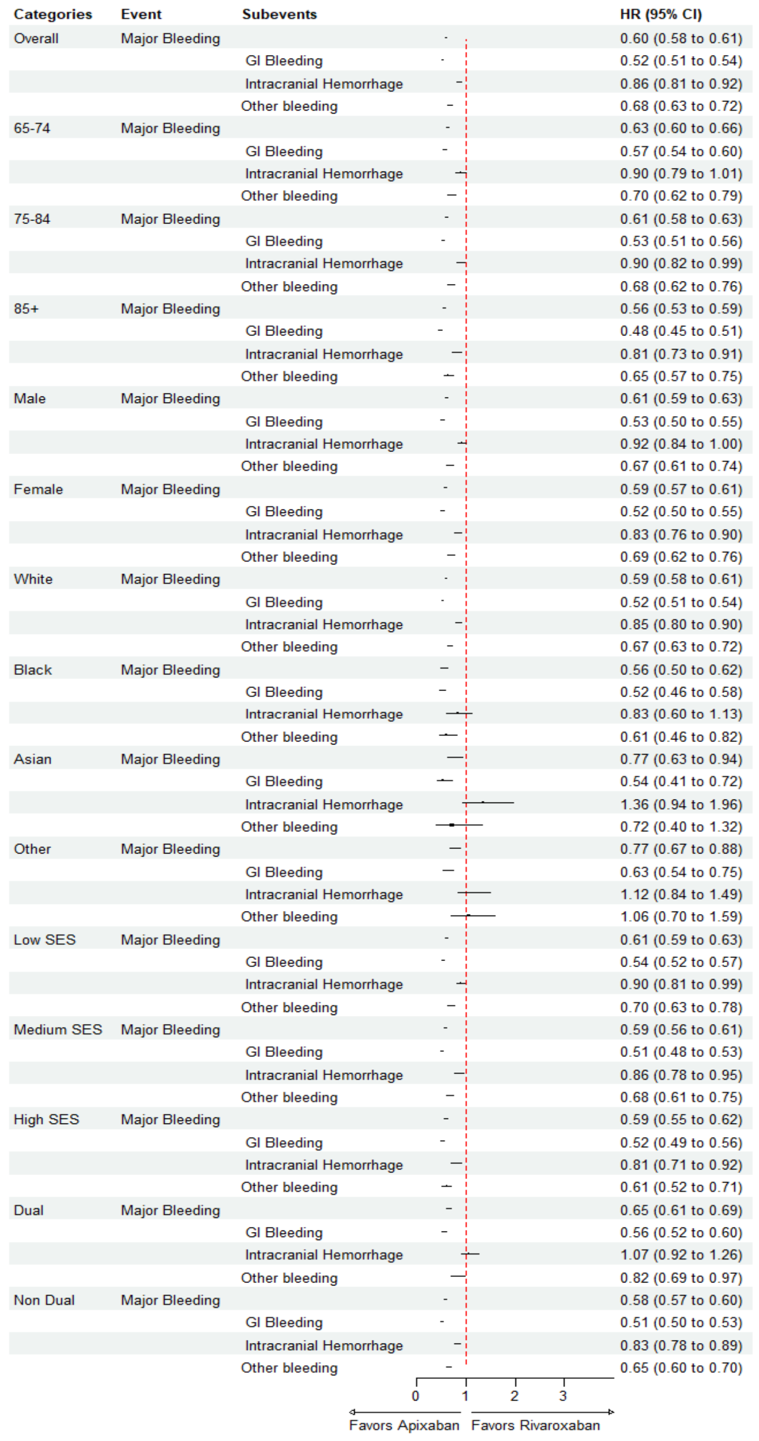

GI: Gastrointestinal; SES: Socioeconomic Status

Figure S5: Comparison between Apixaban vs. Dabigatran on the Risk of stroke/SE among the Overall Population and by Demographic and Socioeconomic Status Subgroups

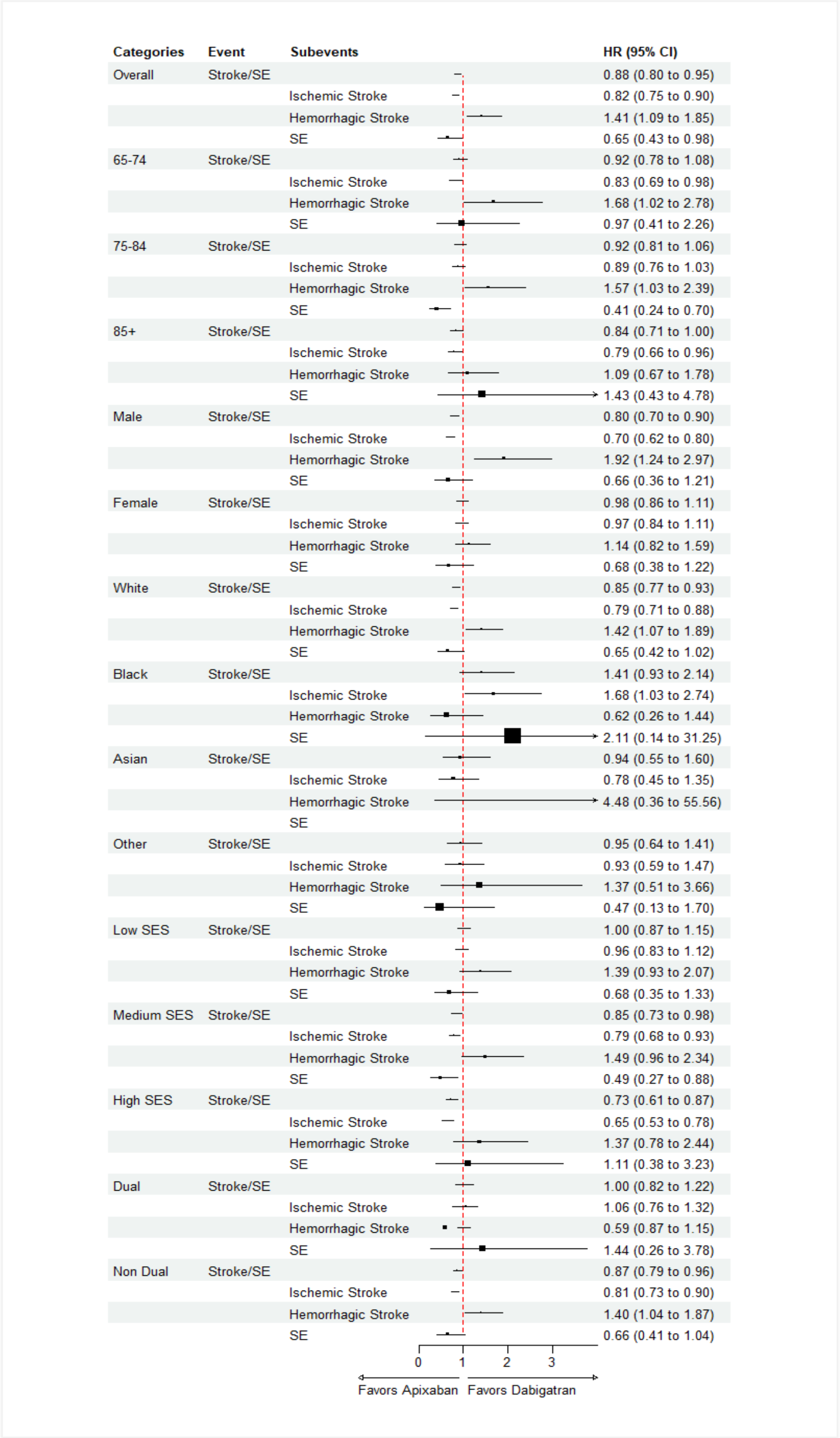

SE: Systemic Embolism; SES: Socioeconomic Status

Figure S6: Comparison between Apixaban vs. Dabigatran on the Risk of Major Bleeding among the Overall Population and by Demographic and Socioeconomic Status Subgroups

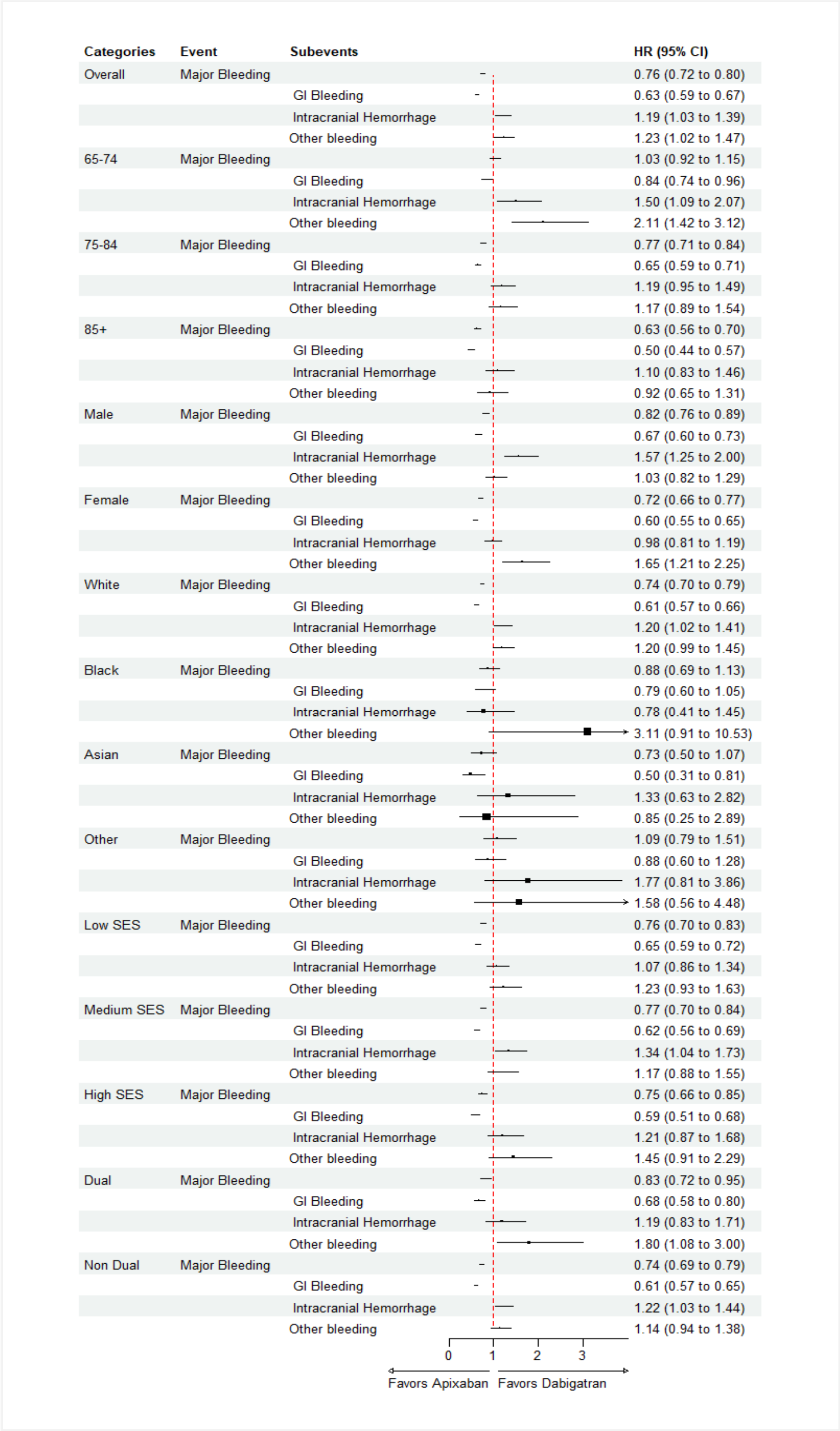

GI: Gastrointestinal; SES: Socioeconomic Status

Figure S7: Comparison between Dabigatran vs. Rivaroxaban on the Risk of stroke/SE among the Overall Population and by Demographic and Socioeconomic Status Subgroups

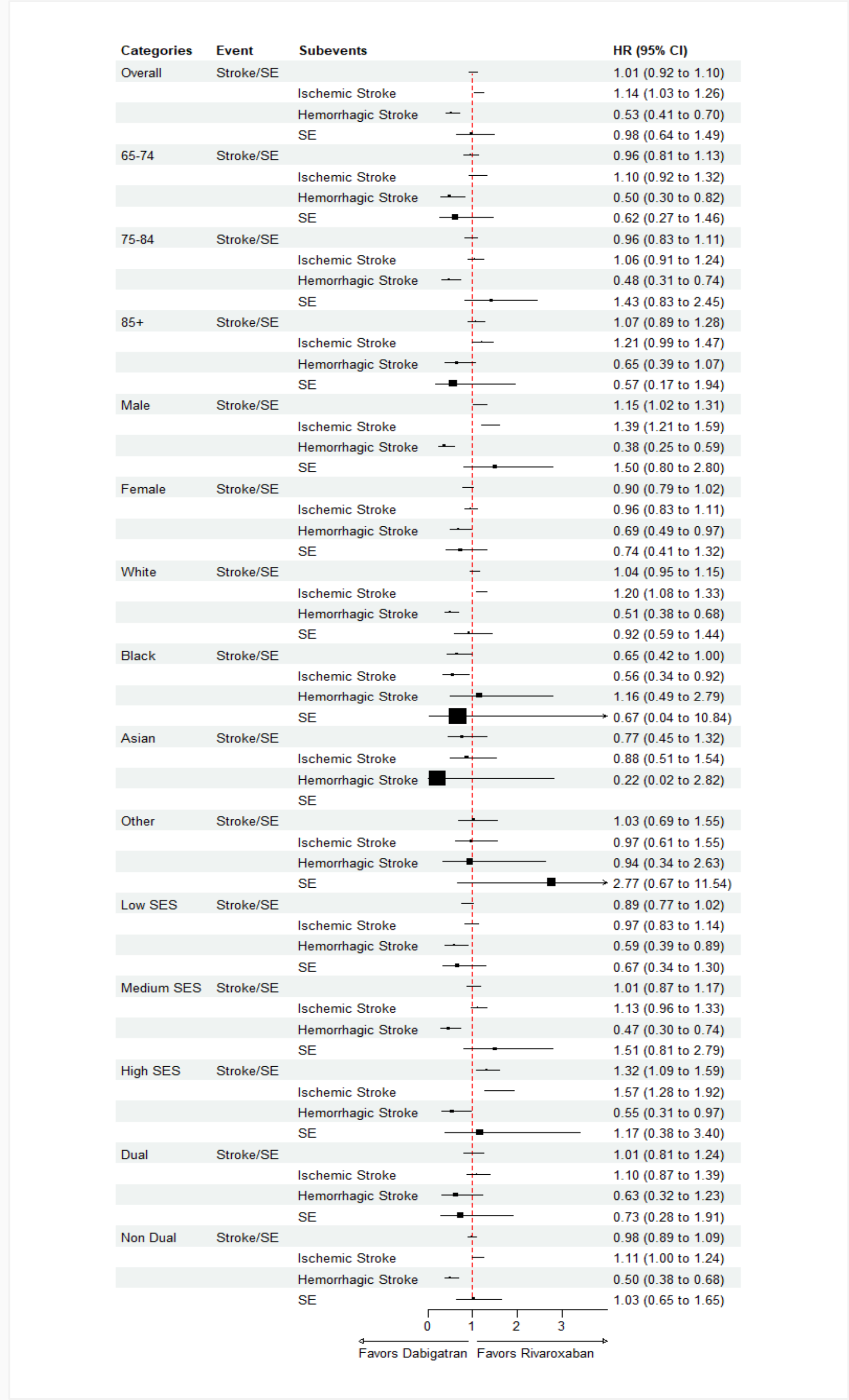

Categories	Event	Subevents	HR (95% CI)
Overall	Stroke/SE		1.01 (0.92 to 1.10)
		Ischemic Stroke	1.14 (1.03 to 1.26)
		Hemorrhagic Stroke	0.53 (0.41 to 0.70)
		SE	0.98 (0.64 to 1.49)
65-74	Stroke/SE		0.96 (0.81 to 1.13)
		Ischemic Stroke	1.10 (0.92 to 1.32)
		Hemorrhagic Stroke	0.50 (0.30 to 0.82)
		SE	0.62 (0.27 to 1.46)
75-84	Stroke/SE		0.96 (0.83 to 1.11)
		Ischemic Stroke	1.06 (0.91 to 1.24)
		Hemorrhagic Stroke	0.48 (0.31 to 0.74)
		SE	1.43 (0.83 to 2.45)
85+	Stroke/SE		1.07 (0.89 to 1.28)
		Ischemic Stroke	1.21 (0.99 to 1.47)
		Hemorrhagic Stroke	0.65 (0.39 to 1.07)
		SE	0.57 (0.17 to 1.94)
Male	Stroke/SE		1.15 (1.02 to 1.31)
		Ischemic Stroke	1.39 (1.21 to 1.59)
		Hemorrhagic Stroke	0.38 (0.25 to 0.59)
		SE	1.50 (0.80 to 2.80)
Female	Stroke/SE		0.90 (0.79 to 1.02)
		Ischemic Stroke	0.96 (0.83 to 1.11)
		Hemorrhagic Stroke	0.69 (0.49 to 0.97)
		SE	0.74 (0.41 to 1.32)
White	Stroke/SE		1.04 (0.95 to 1.15)
		Ischemic Stroke	1.20 (1.08 to 1.33)
		Hemorrhagic Stroke	0.51 (0.38 to 0.68)
		SE	0.92 (0.59 to 1.44)
Black	Stroke/SE		0.65 (0.42 to 1.00)
		Ischemic Stroke	0.56 (0.34 to 0.92)
		Hemorrhagic Stroke	1.16 (0.49 to 2.79)
		SE	0.67 (0.04 to 10.84)
Asian	Stroke/SE		0.77 (0.45 to 1.32)
		Ischemic Stroke	0.88 (0.51 to 1.54)
		Hemorrhagic Stroke	0.22 (0.02 to 2.82)
		SE	
Other	Stroke/SE		1.03 (0.69 to 1.55)
		Ischemic Stroke	0.97 (0.61 to 1.55)
		Hemorrhagic Stroke	0.94 (0.34 to 2.63)
		SE	2.77 (0.67 to 11.54)
Low SES	Stroke/SE		0.89 (0.77 to 1.02)
		Ischemic Stroke	0.97 (0.83 to 1.14)
		Hemorrhagic Stroke	0.59 (0.39 to 0.89)
		SE	0.67 (0.34 to 1.30)
Medium SES	Stroke/SE		1.01 (0.87 to 1.17)
		Ischemic Stroke	1.13 (0.96 to 1.33)
		Hemorrhagic Stroke	0.47 (0.30 to 0.74)
		SE	1.51 (0.81 to 2.79)
High SES	Stroke/SE		1.32 (1.09 to 1.59)
		Ischemic Stroke	1.57 (1.28 to 1.92)
		Hemorrhagic Stroke	0.55 (0.31 to 0.97)
		SE	1.17 (0.38 to 3.40)
Dual	Stroke/SE		1.01 (0.81 to 1.24)
		Ischemic Stroke	1.10 (0.87 to 1.39)
		Hemorrhagic Stroke	0.63 (0.32 to 1.23)
		SE	0.73 (0.28 to 1.91)
Non Dual	Stroke/SE		0.98 (0.89 to 1.09)
		Ischemic Stroke	1.11 (1.00 to 1.24)
		Hemorrhagic Stroke	0.50 (0.38 to 0.68)
		SE	1.03 (0.65 to 1.65)

SE: Systemic Embolism; SES: Socioeconomic Status

Figure S8: Comparison between Dabigatran vs. Rivaroxaban on the Risk of Major Bleeding among the Overall Population and by Demographic and Socioeconomic Status Subgroups

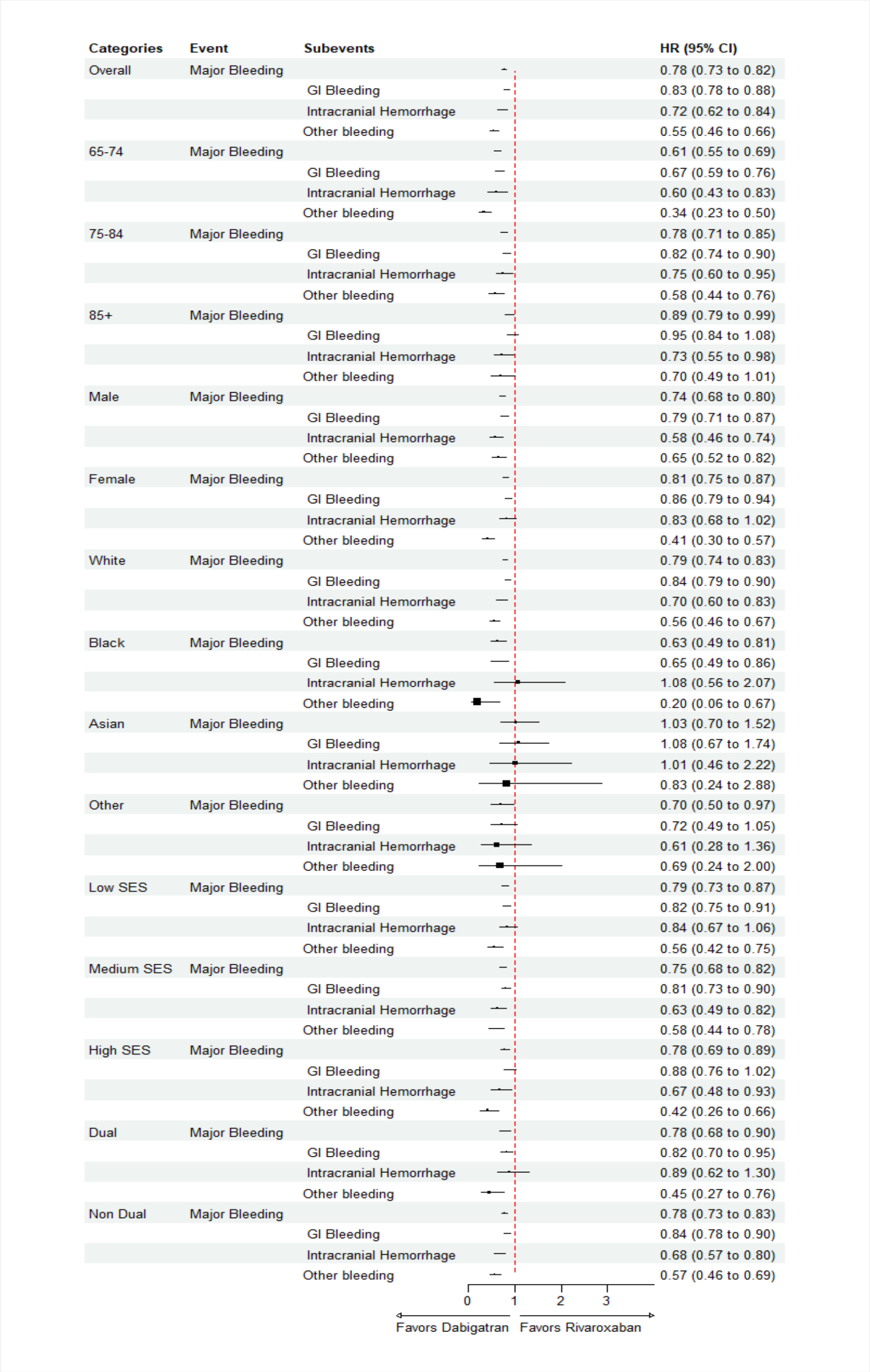

GI: Gastrointestinal; SES: Socioeconomic Status

Figure S9: Comparison between Rivaroxaban vs. Warfarin on the Risk of stroke/SE among the Overall Population and by Demographic and Socioeconomic Status Subgroups

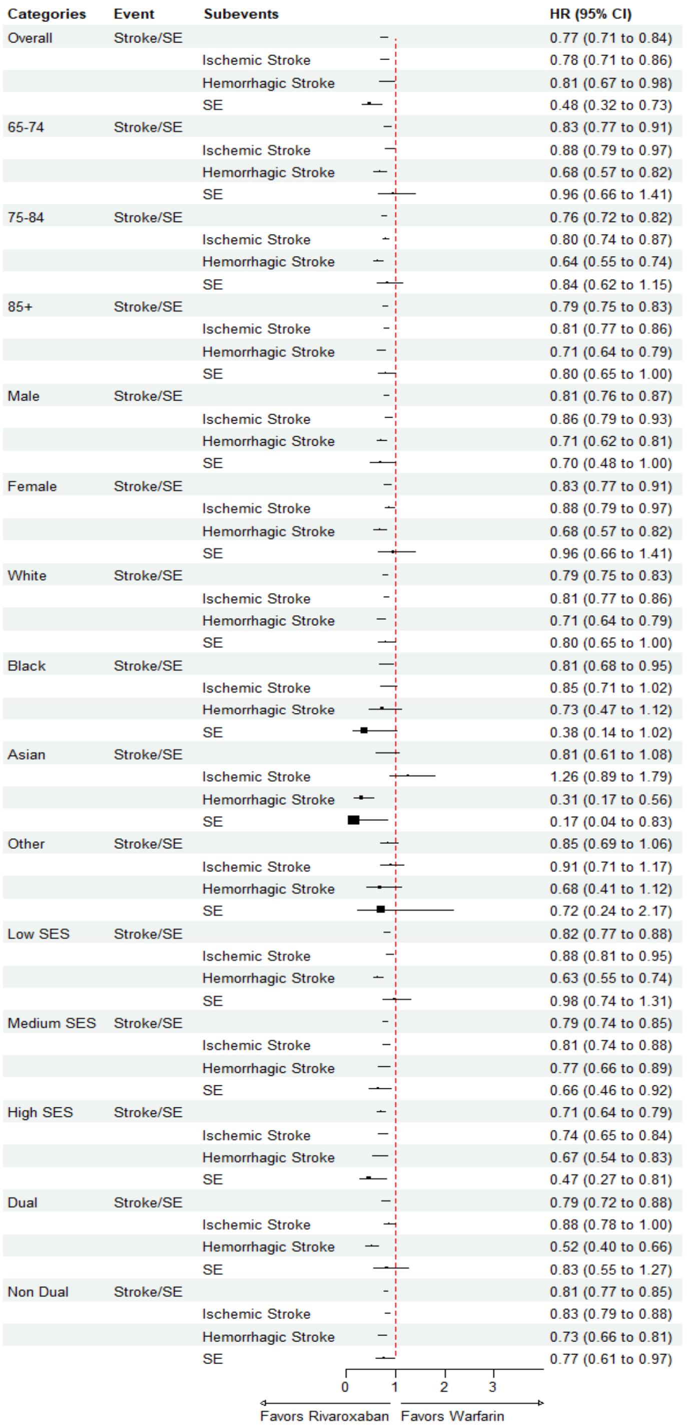

SE: Systemic Embolism; SES: Socioeconomic Status

Figure S10: Comparison between Rivaroxaban vs. Warfarin on the Risk of Major Bleeding among the Overall Population and by Demographic and Socioeconomic Status Subgroups

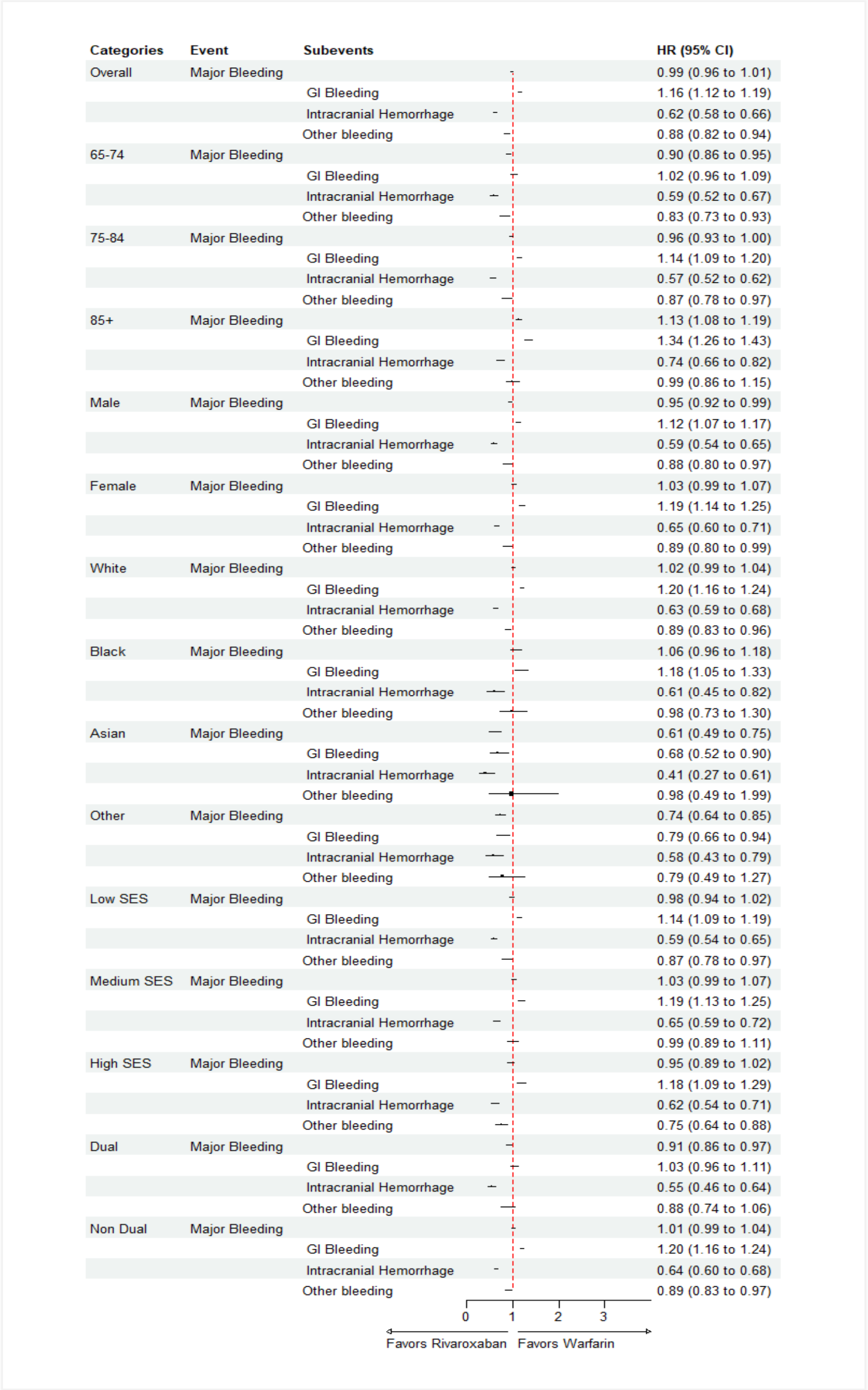

GI: Gastrointestinal; SES: Socioeconomic Status.

Figure S11: Comparison between Dabigatran vs. Warfarin on the Risk of stroke/SE among the Overall Population and by Demographic and Socioeconomic Status Subgroups

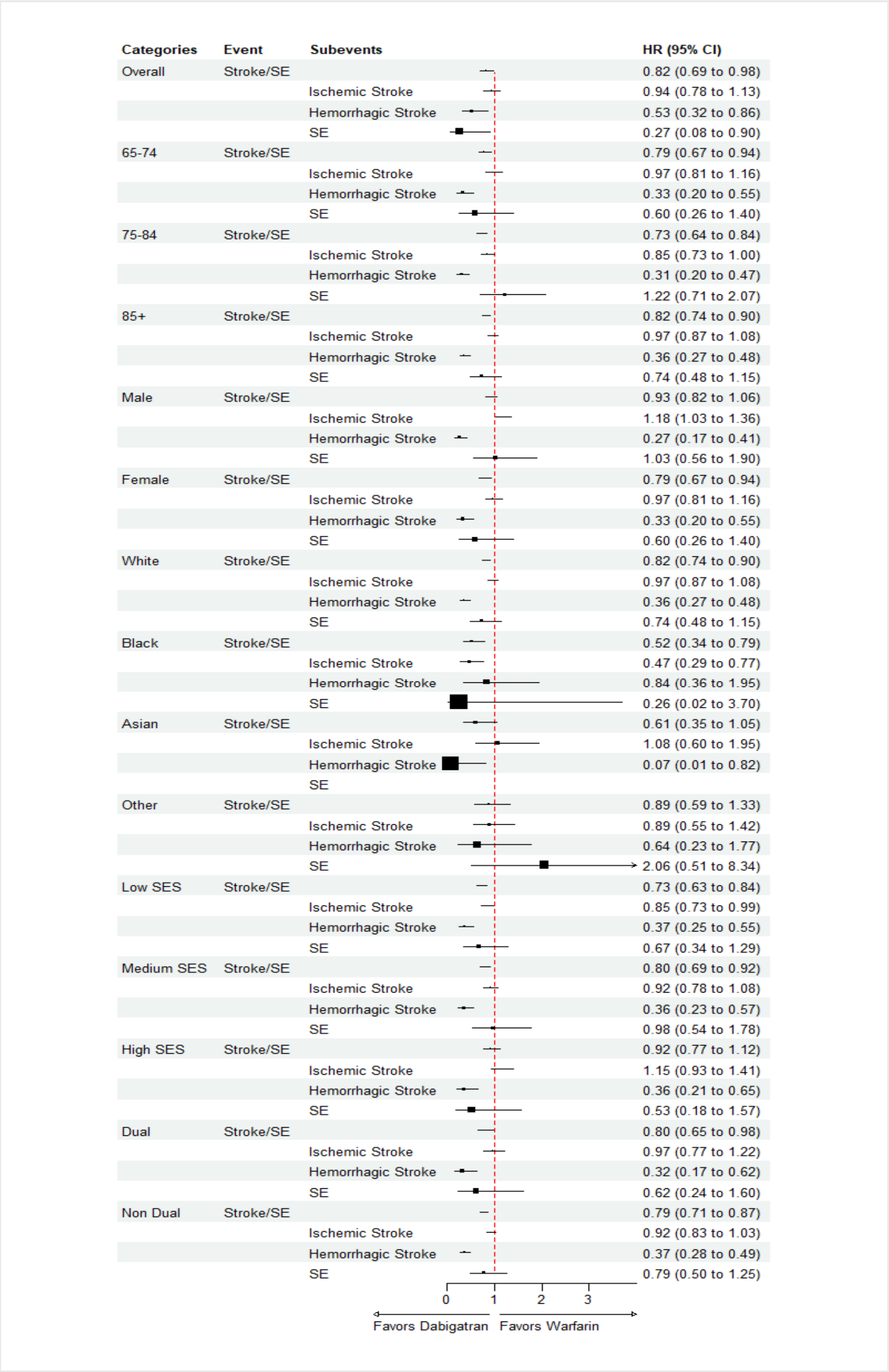

SE: Systemic Embolism; SES: Socioeconomic Status

Figure S12: Comparison between Dabigatran vs. Warfarin on the Risk of Major Bleeding among the Overall Population and by Demographic and Socioeconomic Status Subgroups

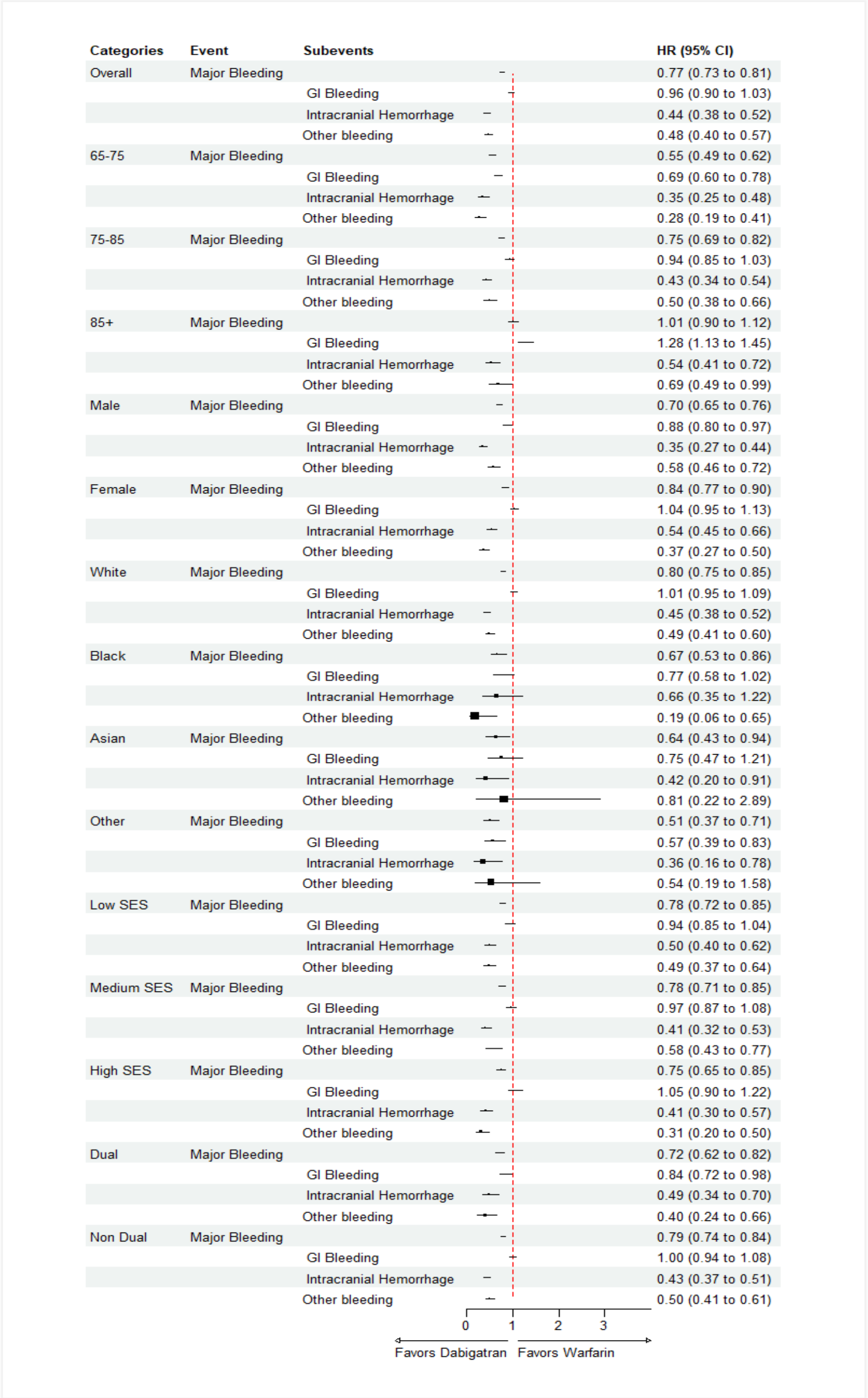

GI: Gastrointestinal; SES: Socioeconomic Status.

Table S1: ICD codes for Atrial fibrillation

ICD-code	ICD 9/10	Description
42731	9	Atrial fibrillation
I480	10	Paroxysmal atrial fibrillation
I481	10	Persistent atrial fibrillation
I4811	10	Longstanding persistent atrial fibrillation
I4819	10	Other persistent atrial fibrillation
I482	10	Chronic atrial fibrillation
I4820	10	Chronic atrial fibrillation, unspecified
I4821	10	Permanent atrial fibrillation
I4891	10	Unspecified atrial fibrillation

Table S2: ICD codes for Stroke Event

ICD-code	ICD 9/10	Stroke Event Type
430	9	Hemorrhagic Stroke
431	9	Hemorrhagic Stroke
432	9	Hemorrhagic Stroke
4320	9	Hemorrhagic Stroke
4321	9	Hemorrhagic Stroke
4329	9	Hemorrhagic Stroke
I6000	10	Hemorrhagic Stroke
I6001	10	Hemorrhagic Stroke
I6002	10	Hemorrhagic Stroke
I6010	10	Hemorrhagic Stroke
I6011	10	Hemorrhagic Stroke
I6012	10	Hemorrhagic Stroke
I602	10	Hemorrhagic Stroke
I6020	10	Hemorrhagic Stroke
I6021	10	Hemorrhagic Stroke
I6022	10	Hemorrhagic Stroke
I6030	10	Hemorrhagic Stroke
I6031	10	Hemorrhagic Stroke
I6032	10	Hemorrhagic Stroke
I604	10	Hemorrhagic Stroke
I6050	10	Hemorrhagic Stroke
I6051	10	Hemorrhagic Stroke
I6052	10	Hemorrhagic Stroke
I606	10	Hemorrhagic Stroke
I607	10	Hemorrhagic Stroke
I608	10	Hemorrhagic Stroke
I609	10	Hemorrhagic Stroke
I610	10	Hemorrhagic Stroke
I611	10	Hemorrhagic Stroke
I612	10	Hemorrhagic Stroke
I613	10	Hemorrhagic Stroke
I614	10	Hemorrhagic Stroke
I615	10	Hemorrhagic Stroke
I616	10	Hemorrhagic Stroke
I618	10	Hemorrhagic Stroke
I619	10	Hemorrhagic Stroke
43301	9	Ischemic Stroke
43311	9	Ischemic Stroke
43321	9	Ischemic Stroke
43331	9	Ischemic Stroke

43381	9	Ischemic Stroke
43391	9	Ischemic Stroke
43401	9	Ischemic Stroke
43411	9	Ischemic Stroke
43491	9	Ischemic Stroke
436	9	Ischemic Stroke
I6300	10	Ischemic Stroke
I63011	10	Ischemic Stroke
I63012	10	Ischemic Stroke
I63013	10	Ischemic Stroke
I63019	10	Ischemic Stroke
I6302	10	Ischemic Stroke
I63031	10	Ischemic Stroke
I63032	10	Ischemic Stroke
I63033	10	Ischemic Stroke
I63039	10	Ischemic Stroke
I6309	10	Ischemic Stroke
I6310	10	Ischemic Stroke
I63111	10	Ischemic Stroke
I63112	10	Ischemic Stroke
I63113	10	Ischemic Stroke
I63119	10	Ischemic Stroke
I6312	10	Ischemic Stroke
I63131	10	Ischemic Stroke
I63132	10	Ischemic Stroke
I63133	10	Ischemic Stroke
I63139	10	Ischemic Stroke
I6319	10	Ischemic Stroke
I6320	10	Ischemic Stroke
I63211	10	Ischemic Stroke
I63212	10	Ischemic Stroke
I63213	10	Ischemic Stroke
I63219	10	Ischemic Stroke
I6322	10	Ischemic Stroke
I63231	10	Ischemic Stroke
I63232	10	Ischemic Stroke
I63233	10	Ischemic Stroke
I63239	10	Ischemic Stroke
I6329	10	Ischemic Stroke
I6330	10	Ischemic Stroke
I63311	10	Ischemic Stroke
I63312	10	Ischemic Stroke
I63313	10	Ischemic Stroke

I63319	10	Ischemic Stroke
I63321	10	Ischemic Stroke
I63322	10	Ischemic Stroke
I63323	10	Ischemic Stroke
I63329	10	Ischemic Stroke
I63331	10	Ischemic Stroke
I63332	10	Ischemic Stroke
I63333	10	Ischemic Stroke
I63339	10	Ischemic Stroke
I63341	10	Ischemic Stroke
I63342	10	Ischemic Stroke
I63343	10	Ischemic Stroke
I63349	10	Ischemic Stroke
I6339	10	Ischemic Stroke
I6340	10	Ischemic Stroke
I63411	10	Ischemic Stroke
I63412	10	Ischemic Stroke
I63413	10	Ischemic Stroke
I63419	10	Ischemic Stroke
I63421	10	Ischemic Stroke
I63422	10	Ischemic Stroke
I63423	10	Ischemic Stroke
I63429	10	Ischemic Stroke
I63431	10	Ischemic Stroke
I63432	10	Ischemic Stroke
I63433	10	Ischemic Stroke
I63439	10	Ischemic Stroke
I63441	10	Ischemic Stroke
I63442	10	Ischemic Stroke
I63443	10	Ischemic Stroke
I63449	10	Ischemic Stroke
I6349	10	Ischemic Stroke
I6350	10	Ischemic Stroke
I63511	10	Ischemic Stroke
I63512	10	Ischemic Stroke
I63513	10	Ischemic Stroke
I63519	10	Ischemic Stroke
I63521	10	Ischemic Stroke
I63522	10	Ischemic Stroke
I63523	10	Ischemic Stroke
I63529	10	Ischemic Stroke
I63531	10	Ischemic Stroke
I63532	10	Ischemic Stroke

I63533	10	Ischemic Stroke
I63539	10	Ischemic Stroke
I63541	10	Ischemic Stroke
I63542	10	Ischemic Stroke
I63543	10	Ischemic Stroke
I63549	10	Ischemic Stroke
I6359	10	Ischemic Stroke
I636	10	Ischemic Stroke
I638	10	Ischemic Stroke
I6381	10	Ischemic Stroke
I6389	10	Ischemic Stroke
I639	10	Ischemic Stroke
I6789	10	Ischemic Stroke
444	9	Systemic Embolism
4440	9	Systemic Embolism
44401	9	Systemic Embolism
44409	9	Systemic Embolism
4441	9	Systemic Embolism
44421	9	Systemic Embolism
44422	9	Systemic Embolism
44481	9	Systemic Embolism
44489	9	Systemic Embolism
4449	9	Systemic Embolism
445	9	Systemic Embolism
44501	9	Systemic Embolism
44502	9	Systemic Embolism
44581	9	Systemic Embolism
44589	9	Systemic Embolism
I7401	10	Systemic Embolism
I7409	10	Systemic Embolism
I7410	10	Systemic Embolism
I7411	10	Systemic Embolism
I7419	10	Systemic Embolism
I742	10	Systemic Embolism
I743	10	Systemic Embolism
I744	10	Systemic Embolism
I745	10	Systemic Embolism
I748	10	Systemic Embolism
I749	10	Systemic Embolism
I75011	10	Systemic Embolism
I75012	10	Systemic Embolism
I75013	10	Systemic Embolism
I75019	10	Systemic Embolism

I75021	10	Systemic Embolism
I75022	10	Systemic Embolism
I75023	10	Systemic Embolism
I75029	10	Systemic Embolism
I7581	10	Systemic Embolism
I7589	10	Systemic Embolism

Table S3: ICD codes for Bleeding Events

ICD-Code	ICD-9/10	Bleed Event Type
4560	9	Major Gastrointestinal bleeding event
45620	9	Major Gastrointestinal bleeding event
53082	9	Major Gastrointestinal bleeding event
5310	9	Major Gastrointestinal bleeding event
53100	9	Major Gastrointestinal bleeding event
53101	9	Major Gastrointestinal bleeding event
5312	9	Major Gastrointestinal bleeding event
53120	9	Major Gastrointestinal bleeding event
53121	9	Major Gastrointestinal bleeding event
5314	9	Major Gastrointestinal bleeding event
53140	9	Major Gastrointestinal bleeding event
53141	9	Major Gastrointestinal bleeding event
5316	9	Major Gastrointestinal bleeding event
53160	9	Major Gastrointestinal bleeding event
53161	9	Major Gastrointestinal bleeding event
5320	9	Major Gastrointestinal bleeding event
53200	9	Major Gastrointestinal bleeding event
53201	9	Major Gastrointestinal bleeding event
5322	9	Major Gastrointestinal bleeding event
53220	9	Major Gastrointestinal bleeding event
53221	9	Major Gastrointestinal bleeding event
5324	9	Major Gastrointestinal bleeding event
53240	9	Major Gastrointestinal bleeding event
53241	9	Major Gastrointestinal bleeding event
5326	9	Major Gastrointestinal bleeding event
53260	9	Major Gastrointestinal bleeding event
53261	9	Major Gastrointestinal bleeding event
5330	9	Major Gastrointestinal bleeding event
53300	9	Major Gastrointestinal bleeding event
53301	9	Major Gastrointestinal bleeding event
5332	9	Major Gastrointestinal bleeding event
53320	9	Major Gastrointestinal bleeding event
53321	9	Major Gastrointestinal bleeding event
5334	9	Major Gastrointestinal bleeding event
53340	9	Major Gastrointestinal bleeding event
53341	9	Major Gastrointestinal bleeding event
5336	9	Major Gastrointestinal bleeding event
53360	9	Major Gastrointestinal bleeding event
53361	9	Major Gastrointestinal bleeding event
5340	9	Major Gastrointestinal bleeding event

53400	9	Major Gastrointestinal bleeding event
53401	9	Major Gastrointestinal bleeding event
5342	9	Major Gastrointestinal bleeding event
53420	9	Major Gastrointestinal bleeding event
53421	9	Major Gastrointestinal bleeding event
5344	9	Major Gastrointestinal bleeding event
53440	9	Major Gastrointestinal bleeding event
53441	9	Major Gastrointestinal bleeding event
5346	9	Major Gastrointestinal bleeding event
53460	9	Major Gastrointestinal bleeding event
53461	9	Major Gastrointestinal bleeding event
53501	9	Major Gastrointestinal bleeding event
53511	9	Major Gastrointestinal bleeding event
53521	9	Major Gastrointestinal bleeding event
53531	9	Major Gastrointestinal bleeding event
53541	9	Major Gastrointestinal bleeding event
53551	9	Major Gastrointestinal bleeding event
53561	9	Major Gastrointestinal bleeding event
53783	9	Major Gastrointestinal bleeding event
56202	9	Major Gastrointestinal bleeding event
56203	9	Major Gastrointestinal bleeding event
56212	9	Major Gastrointestinal bleeding event
56213	9	Major Gastrointestinal bleeding event
56881	9	Major Gastrointestinal bleeding event
5693	9	Major Gastrointestinal bleeding event
56985	9	Major Gastrointestinal bleeding event
578	9	Major Gastrointestinal bleeding event
5780	9	Major Gastrointestinal bleeding event
5781	9	Major Gastrointestinal bleeding event
5789	9	Major Gastrointestinal bleeding event
I8501	10	Major Gastrointestinal bleeding event
I8511	10	Major Gastrointestinal bleeding event
K226	10	Major Gastrointestinal bleeding event
K250	10	Major Gastrointestinal bleeding event
K252	10	Major Gastrointestinal bleeding event
K254	10	Major Gastrointestinal bleeding event
K256	10	Major Gastrointestinal bleeding event
K260	10	Major Gastrointestinal bleeding event
K262	10	Major Gastrointestinal bleeding event
K264	10	Major Gastrointestinal bleeding event
K266	10	Major Gastrointestinal bleeding event
K270	10	Major Gastrointestinal bleeding event
K272	10	Major Gastrointestinal bleeding event

K274	10	Major Gastrointestinal bleeding event
K276	10	Major Gastrointestinal bleeding event
K280	10	Major Gastrointestinal bleeding event
K282	10	Major Gastrointestinal bleeding event
K284	10	Major Gastrointestinal bleeding event
K286	10	Major Gastrointestinal bleeding event
K2901	10	Major Gastrointestinal bleeding event
K2921	10	Major Gastrointestinal bleeding event
K2931	10	Major Gastrointestinal bleeding event
K2941	10	Major Gastrointestinal bleeding event
K2951	10	Major Gastrointestinal bleeding event
K2961	10	Major Gastrointestinal bleeding event
K2971	10	Major Gastrointestinal bleeding event
K2981	10	Major Gastrointestinal bleeding event
K2991	10	Major Gastrointestinal bleeding event
K31811	10	Major Gastrointestinal bleeding event
K3182	10	Major Gastrointestinal bleeding event
K5521	10	Major Gastrointestinal bleeding event
K5701	10	Major Gastrointestinal bleeding event
K5711	10	Major Gastrointestinal bleeding event
K5713	10	Major Gastrointestinal bleeding event
K5721	10	Major Gastrointestinal bleeding event
K5731	10	Major Gastrointestinal bleeding event
K5733	10	Major Gastrointestinal bleeding event
K5741	10	Major Gastrointestinal bleeding event
K5751	10	Major Gastrointestinal bleeding event
K5753	10	Major Gastrointestinal bleeding event
K5781	10	Major Gastrointestinal bleeding event
K5791	10	Major Gastrointestinal bleeding event
K5793	10	Major Gastrointestinal bleeding event
K625	10	Major Gastrointestinal bleeding event
K6381	10	Major Gastrointestinal bleeding event
K661	10	Major Gastrointestinal bleeding event
K920	10	Major Gastrointestinal bleeding event
K921	10	Major Gastrointestinal bleeding event
K922	10	Major Gastrointestinal bleeding event
K9161	10	Major Gastrointestinal bleeding event
K9162	10	Major Gastrointestinal bleeding event
K91840	10	Major Gastrointestinal bleeding event
K91841	10	Major Gastrointestinal bleeding event
430	9	Major Intracranial Hemorrhage (ICH)
431	9	Major Intracranial Hemorrhage (ICH)
4320	9	Major Intracranial Hemorrhage (ICH)

4321	9	Major Intracranial Hemorrhage (ICH)
4329	9	Major Intracranial Hemorrhage (ICH)
8520	9	Major Intracranial Hemorrhage (ICH)
85200	9	Major Intracranial Hemorrhage (ICH)
85201	9	Major Intracranial Hemorrhage (ICH)
85202	9	Major Intracranial Hemorrhage (ICH)
85203	9	Major Intracranial Hemorrhage (ICH)
85204	9	Major Intracranial Hemorrhage (ICH)
85205	9	Major Intracranial Hemorrhage (ICH)
85206	9	Major Intracranial Hemorrhage (ICH)
85209	9	Major Intracranial Hemorrhage (ICH)
8522	9	Major Intracranial Hemorrhage (ICH)
85220	9	Major Intracranial Hemorrhage (ICH)
85221	9	Major Intracranial Hemorrhage (ICH)
85222	9	Major Intracranial Hemorrhage (ICH)
85223	9	Major Intracranial Hemorrhage (ICH)
85224	9	Major Intracranial Hemorrhage (ICH)
85225	9	Major Intracranial Hemorrhage (ICH)
85226	9	Major Intracranial Hemorrhage (ICH)
85229	9	Major Intracranial Hemorrhage (ICH)
8524	9	Major Intracranial Hemorrhage (ICH)
85240	9	Major Intracranial Hemorrhage (ICH)
85241	9	Major Intracranial Hemorrhage (ICH)
85242	9	Major Intracranial Hemorrhage (ICH)
85243	9	Major Intracranial Hemorrhage (ICH)
85244	9	Major Intracranial Hemorrhage (ICH)
85245	9	Major Intracranial Hemorrhage (ICH)
85246	9	Major Intracranial Hemorrhage (ICH)
85249	9	Major Intracranial Hemorrhage (ICH)
8530	9	Major Intracranial Hemorrhage (ICH)
85300	9	Major Intracranial Hemorrhage (ICH)
85301	9	Major Intracranial Hemorrhage (ICH)
85302	9	Major Intracranial Hemorrhage (ICH)
85303	9	Major Intracranial Hemorrhage (ICH)
85304	9	Major Intracranial Hemorrhage (ICH)
85305	9	Major Intracranial Hemorrhage (ICH)
85306	9	Major Intracranial Hemorrhage (ICH)
85309	9	Major Intracranial Hemorrhage (ICH)
I6000	10	Major Intracranial Hemorrhage (ICH)
I6001	10	Major Intracranial Hemorrhage (ICH)
I6002	10	Major Intracranial Hemorrhage (ICH)
I6010	10	Major Intracranial Hemorrhage (ICH)
I6011	10	Major Intracranial Hemorrhage (ICH)

I6012	10	Major Intracranial Hemorrhage (ICH)
I602	10	Major Intracranial Hemorrhage (ICH)
I6020	10	Major Intracranial Hemorrhage (ICH)
I6021	10	Major Intracranial Hemorrhage (ICH)
I6022	10	Major Intracranial Hemorrhage (ICH)
I6030	10	Major Intracranial Hemorrhage (ICH)
I6031	10	Major Intracranial Hemorrhage (ICH)
I6032	10	Major Intracranial Hemorrhage (ICH)
I6050	10	Major Intracranial Hemorrhage (ICH)
I6051	10	Major Intracranial Hemorrhage (ICH)
I6052	10	Major Intracranial Hemorrhage (ICH)
I606	10	Major Intracranial Hemorrhage (ICH)
I607	10	Major Intracranial Hemorrhage (ICH)
I608	10	Major Intracranial Hemorrhage (ICH)
I609	10	Major Intracranial Hemorrhage (ICH)
I610	10	Major Intracranial Hemorrhage (ICH)
I611	10	Major Intracranial Hemorrhage (ICH)
I612	10	Major Intracranial Hemorrhage (ICH)
I613	10	Major Intracranial Hemorrhage (ICH)
I614	10	Major Intracranial Hemorrhage (ICH)
I615	10	Major Intracranial Hemorrhage (ICH)
I616	10	Major Intracranial Hemorrhage (ICH)
I618	10	Major Intracranial Hemorrhage (ICH)
I619	10	Major Intracranial Hemorrhage (ICH)
I6200	10	Major Intracranial Hemorrhage (ICH)
I6201	10	Major Intracranial Hemorrhage (ICH)
I6202	10	Major Intracranial Hemorrhage (ICH)
I6203	10	Major Intracranial Hemorrhage (ICH)
I621	10	Major Intracranial Hemorrhage (ICH)
I629	10	Major Intracranial Hemorrhage (ICH)
S06340A	10	Major Intracranial Hemorrhage (ICH)
S06341A	10	Major Intracranial Hemorrhage (ICH)
S06342A	10	Major Intracranial Hemorrhage (ICH)
S06343A	10	Major Intracranial Hemorrhage (ICH)
S06344A	10	Major Intracranial Hemorrhage (ICH)
S06345A	10	Major Intracranial Hemorrhage (ICH)
S06346A	10	Major Intracranial Hemorrhage (ICH)
S06347A	10	Major Intracranial Hemorrhage (ICH)
S06348A	10	Major Intracranial Hemorrhage (ICH)
S06349A	10	Major Intracranial Hemorrhage (ICH)
S06350A	10	Major Intracranial Hemorrhage (ICH)
S06351A	10	Major Intracranial Hemorrhage (ICH)
S06352A	10	Major Intracranial Hemorrhage (ICH)

S06353A	10	Major Intracranial Hemorrhage (ICH)
S06354A	10	Major Intracranial Hemorrhage (ICH)
S06355A	10	Major Intracranial Hemorrhage (ICH)
S06356A	10	Major Intracranial Hemorrhage (ICH)
S06357A	10	Major Intracranial Hemorrhage (ICH)
S06358A	10	Major Intracranial Hemorrhage (ICH)
S06359A	10	Major Intracranial Hemorrhage (ICH)
S06360A	10	Major Intracranial Hemorrhage (ICH)
S06361A	10	Major Intracranial Hemorrhage (ICH)
S06362A	10	Major Intracranial Hemorrhage (ICH)
S06363A	10	Major Intracranial Hemorrhage (ICH)
S06364A	10	Major Intracranial Hemorrhage (ICH)
S06365A	10	Major Intracranial Hemorrhage (ICH)
S06366A	10	Major Intracranial Hemorrhage (ICH)
S06367A	10	Major Intracranial Hemorrhage (ICH)
S06368A	10	Major Intracranial Hemorrhage (ICH)
S06369A	10	Major Intracranial Hemorrhage (ICH)
S064X0A	10	Major Intracranial Hemorrhage (ICH)
S064X1A	10	Major Intracranial Hemorrhage (ICH)
S064X2A	10	Major Intracranial Hemorrhage (ICH)
S064X3A	10	Major Intracranial Hemorrhage (ICH)
S064X4A	10	Major Intracranial Hemorrhage (ICH)
S064X5A	10	Major Intracranial Hemorrhage (ICH)
S064X6A	10	Major Intracranial Hemorrhage (ICH)
S064X7A	10	Major Intracranial Hemorrhage (ICH)
S064X8A	10	Major Intracranial Hemorrhage (ICH)
S064X9A	10	Major Intracranial Hemorrhage (ICH)
S065X0A	10	Major Intracranial Hemorrhage (ICH)
S065X1A	10	Major Intracranial Hemorrhage (ICH)
S065X2A	10	Major Intracranial Hemorrhage (ICH)
S065X3A	10	Major Intracranial Hemorrhage (ICH)
S065X4A	10	Major Intracranial Hemorrhage (ICH)
S065X5A	10	Major Intracranial Hemorrhage (ICH)
S065X6A	10	Major Intracranial Hemorrhage (ICH)
S065X7A	10	Major Intracranial Hemorrhage (ICH)
S065X8A	10	Major Intracranial Hemorrhage (ICH)
S065X9A	10	Major Intracranial Hemorrhage (ICH)
S066X0A	10	Major Intracranial Hemorrhage (ICH)
S066X1A	10	Major Intracranial Hemorrhage (ICH)
S066X2A	10	Major Intracranial Hemorrhage (ICH)
S066X3A	10	Major Intracranial Hemorrhage (ICH)
S066X4A	10	Major Intracranial Hemorrhage (ICH)
S066X5A	10	Major Intracranial Hemorrhage (ICH)

S066X6A	10	Major Intracranial Hemorrhage (ICH)
S066X7A	10	Major Intracranial Hemorrhage (ICH)
S066X8A	10	Major Intracranial Hemorrhage (ICH)
S066X9A	10	Major Intracranial Hemorrhage (ICH)
36043	9	Major Other hemorrhage
36243	9	Major Other hemorrhage
36281	9	Major Other hemorrhage
36361	9	Major Other hemorrhage
36362	9	Major Other hemorrhage
36372	9	Major Other hemorrhage
36441	9	Major Other hemorrhage
37272	9	Major Other hemorrhage
37481	9	Major Other hemorrhage
37632	9	Major Other hemorrhage
37742	9	Major Other hemorrhage
37923	9	Major Other hemorrhage
4230	9	Major Other hemorrhage
5967	9	Major Other hemorrhage
5997	9	Major Other hemorrhage
59970	9	Major Other hemorrhage
59971	9	Major Other hemorrhage
59972	9	Major Other hemorrhage
6021	9	Major Other hemorrhage
6201	9	Major Other hemorrhage
6214	9	Major Other hemorrhage
6262	9	Major Other hemorrhage
6265	9	Major Other hemorrhage
6267	9	Major Other hemorrhage
6268	9	Major Other hemorrhage
6269	9	Major Other hemorrhage
7191	9	Major Other hemorrhage
71910	9	Major Other hemorrhage
71911	9	Major Other hemorrhage
71912	9	Major Other hemorrhage
71913	9	Major Other hemorrhage
71914	9	Major Other hemorrhage
71915	9	Major Other hemorrhage
71916	9	Major Other hemorrhage
71917	9	Major Other hemorrhage
71918	9	Major Other hemorrhage
71919	9	Major Other hemorrhage
7827	9	Major Other hemorrhage
7847	9	Major Other hemorrhage

7848	9	Major Other hemorrhage
7863	9	Major Other hemorrhage
78630	9	Major Other hemorrhage
78631	9	Major Other hemorrhage
78639	9	Major Other hemorrhage
9582	9	Major Other hemorrhage
99702	9	Major Other hemorrhage
99811	9	Major Other hemorrhage
D62	10	Major Other hemorrhage
D7801	10	Major Other hemorrhage
D7802	10	Major Other hemorrhage
D7821	10	Major Other hemorrhage
D7822	10	Major Other hemorrhage
E3601	10	Major Other hemorrhage
E3602	10	Major Other hemorrhage
E89810	10	Major Other hemorrhage
E89811	10	Major Other hemorrhage
G9731	10	Major Other hemorrhage
G9732	10	Major Other hemorrhage
G9751	10	Major Other hemorrhage
G9752	10	Major Other hemorrhage
H05231	10	Major Other hemorrhage
H05232	10	Major Other hemorrhage
H05233	10	Major Other hemorrhage
H05239	10	Major Other hemorrhage
H1130	10	Major Other hemorrhage
H1131	10	Major Other hemorrhage
H1132	10	Major Other hemorrhage
H1133	10	Major Other hemorrhage
H2100	10	Major Other hemorrhage
H2101	10	Major Other hemorrhage
H2102	10	Major Other hemorrhage
H2103	10	Major Other hemorrhage
H31301	10	Major Other hemorrhage
H31302	10	Major Other hemorrhage
H31303	10	Major Other hemorrhage
H31309	10	Major Other hemorrhage
H31311	10	Major Other hemorrhage
H31312	10	Major Other hemorrhage
H31313	10	Major Other hemorrhage
H31319	10	Major Other hemorrhage
H31411	10	Major Other hemorrhage
H31412	10	Major Other hemorrhage

H31413	10	Major Other hemorrhage
H31419	10	Major Other hemorrhage
H3560	10	Major Other hemorrhage
H3561	10	Major Other hemorrhage
H3562	10	Major Other hemorrhage
H3563	10	Major Other hemorrhage
H35731	10	Major Other hemorrhage
H35732	10	Major Other hemorrhage
H35733	10	Major Other hemorrhage
H35739	10	Major Other hemorrhage
H4310	10	Major Other hemorrhage
H4311	10	Major Other hemorrhage
H4312	10	Major Other hemorrhage
H4313	10	Major Other hemorrhage
H44811	10	Major Other hemorrhage
H44812	10	Major Other hemorrhage
H44813	10	Major Other hemorrhage
H44819	10	Major Other hemorrhage
H47021	10	Major Other hemorrhage
H47022	10	Major Other hemorrhage
H47023	10	Major Other hemorrhage
H47029	10	Major Other hemorrhage
H59111	10	Major Other hemorrhage
H59112	10	Major Other hemorrhage
H59113	10	Major Other hemorrhage
H59119	10	Major Other hemorrhage
H59121	10	Major Other hemorrhage
H59122	10	Major Other hemorrhage
H59123	10	Major Other hemorrhage
H59129	10	Major Other hemorrhage
H59311	10	Major Other hemorrhage
H59312	10	Major Other hemorrhage
H59313	10	Major Other hemorrhage
H59319	10	Major Other hemorrhage
H59321	10	Major Other hemorrhage
H59322	10	Major Other hemorrhage
H59323	10	Major Other hemorrhage
H59329	10	Major Other hemorrhage
H9521	10	Major Other hemorrhage
H9522	10	Major Other hemorrhage
H9541	10	Major Other hemorrhage
H9542	10	Major Other hemorrhage
I312	10	Major Other hemorrhage

I97410	10	Major Other hemorrhage
I97411	10	Major Other hemorrhage
I97418	10	Major Other hemorrhage
I9742	10	Major Other hemorrhage
I97610	10	Major Other hemorrhage
I97611	10	Major Other hemorrhage
I97618	10	Major Other hemorrhage
I97620	10	Major Other hemorrhage
J9561	10	Major Other hemorrhage
J9562	10	Major Other hemorrhage
J95830	10	Major Other hemorrhage
J95831	10	Major Other hemorrhage
L7601	10	Major Other hemorrhage
L7602	10	Major Other hemorrhage
L7621	10	Major Other hemorrhage
L7622	10	Major Other hemorrhage
M2500	10	Major Other hemorrhage
M25011	10	Major Other hemorrhage
M25012	10	Major Other hemorrhage
M25019	10	Major Other hemorrhage
M25021	10	Major Other hemorrhage
M25022	10	Major Other hemorrhage
M25029	10	Major Other hemorrhage
M25031	10	Major Other hemorrhage
M25032	10	Major Other hemorrhage
M25039	10	Major Other hemorrhage
M25041	10	Major Other hemorrhage
M25042	10	Major Other hemorrhage
M25049	10	Major Other hemorrhage
M25051	10	Major Other hemorrhage
M25052	10	Major Other hemorrhage
M25059	10	Major Other hemorrhage
M25061	10	Major Other hemorrhage
M25062	10	Major Other hemorrhage
M25069	10	Major Other hemorrhage
M25071	10	Major Other hemorrhage
M25072	10	Major Other hemorrhage
M25073	10	Major Other hemorrhage
M25074	10	Major Other hemorrhage
M25075	10	Major Other hemorrhage
M25076	10	Major Other hemorrhage
M2508	10	Major Other hemorrhage
M96810	10	Major Other hemorrhage

M96811	10	Major Other hemorrhage
M96830	10	Major Other hemorrhage
M96831	10	Major Other hemorrhage
N421	10	Major Other hemorrhage
N857	10	Major Other hemorrhage
N897	10	Major Other hemorrhage
N920	10	Major Other hemorrhage
N923	10	Major Other hemorrhage
N930	10	Major Other hemorrhage
N938	10	Major Other hemorrhage
N939	10	Major Other hemorrhage
N9961	10	Major Other hemorrhage
N9962	10	Major Other hemorrhage
N99820	10	Major Other hemorrhage
N99821	10	Major Other hemorrhage
R040	10	Major Other hemorrhage
R041	10	Major Other hemorrhage
R042	10	Major Other hemorrhage
R0489	10	Major Other hemorrhage
R049	10	Major Other hemorrhage
R233	10	Major Other hemorrhage
R310	10	Major Other hemorrhage
R319	10	Major Other hemorrhage
R58	10	Major Other hemorrhage
T792XXA	10	Major Other hemorrhage
30230N1	10 PCS	Major Other hemorrhage
30230P1	10 PCS	Major Other hemorrhage
30233N1	10 PCS	Major Other hemorrhage
30233P1	10 PCS	Major Other hemorrhage
30240N1	10 PCS	Major Other hemorrhage
30233P1	10 PCS	Major Other hemorrhage
30240N1	10 PCS	Major Other hemorrhage
30240P1	10 PCS	Major Other hemorrhage
30243N1	10 PCS	Major Other hemorrhage
30243P1	10 PCS	Major Other hemorrhage
30250N1	10 PCS	Major Other hemorrhage
30250P1	10 PCS	Major Other hemorrhage
30253N1	10 PCS	Major Other hemorrhage
30253P1	10 PCS	Major Other hemorrhage
30260N1	10 PCS	Major Other hemorrhage
30260P1	10 PCS	Major Other hemorrhage
30263N1	10 PCS	Major Other hemorrhage

30263P1	10 PCS	Major Other hemorrhage
9904	9 PCS	Major Other hemorrhage
4443	9 PCS	Major GI Bleed
